# Phenotypic and Molecular Characterization of Adult Plant Resistance to Stripe Rust (*Puccinia striiformis* f. sp. *tritici*) in Exotic and Indigenous Wheat Germplasm

**DOI:** 10.3390/plants15213315

**Published:** 2026-10-26

**Authors:** Ankit Kumar Singh, Prem Lal Kashyap, Sudheer Kumar, Rajender Singh Beniwal, Vikas Gupta, Satish Kumar, Partiban Manikannan, Arun Gupta, Om Prakash Gangwar, Rajender Singh, Pradeep Sharma, Ratan Tiwari

**Affiliations:** 1 ICAR-Indian Institute of Wheat and Barley Research, Karnal 132001, Haryana, India; ankitsinghupc24@gmail.com (A.K.S.);; 2 Department of Plant Pathology, College of Agriculture, Chaudhary Charan Singh Haryana Agricultural University, Hisar 125001, Haryana, India

**Keywords:** *Triticum aestivum*, *Puccinia striiformis* f. sp. *tritici*, adult plant resistance, *Yr18*, *Yr29*, *Yr46*, gene pyramiding, SSR markers, slow rusting

## Abstract

Stripe rust, caused by *Puccinia striiformis* f. sp. *tritici* (Pst), is one of the most destructive diseases of wheat (*Triticum aestivum* L.) worldwide. The frequent breakdown of race-specific, all-stage resistance genes due to the emergence of new virulent pathotypes underscores the need for durable resistance. Adult plant resistance (APR) genes confer partial, largely race-non-specific resistance and provide a sustainable strategy for long-term disease management. In this study, two hundred exotic and indigenous wheat genotypes were evaluated for stripe rust resistance at the seedling stage under controlled glasshouse conditions using five predominant Indian Pst pathotypes (46S119, 110S119, 238S119, 110S84, and 47S103), and at the adult-plant stage in field trials conducted over three consecutive crop seasons (2021–2024). Disease response was assessed using latent period, lesion length, coefficient of infection (CI), final disease severity (FDS), and area under the disease progress curve (AUDPC). Gene-linked SSR markers (*csLV34*, *wmc44*, and *gwm192*) were employed to detect the APR genes *Yr18*, *Yr29*, and *Yr46*, respectively. Field evaluation classified eight genotypes as immune, nine as highly resistant, 22 as resistant, 58 as moderately resistant, 14 as moderately susceptible, 61 as susceptible, and 28 as highly susceptible. Marker analysis identified *Yr18*, *Yr29*, and *Yr46* individually in 9, 38, and 36 genotypess, respectively, while 20, 10, 51, and 27 genotypes carried the *Yr18*+*Yr29*, *Yr18*+*Yr46*, *Yr29*+*Yr46*, and *Yr18*+*Yr29*+*Yr46* combinations, respectively; only nine genotypes lacked all three markers. Principal component and correlation analyses revealed that increasing APR gene pyramiding was significantly associated with prolonged latent period and reduced lesion length, CI, and AUDPC, demonstrating additive effects on resistance. Notably, eight genotypes, including BW 35109, BW 35110, BW 35112, BW 35114, BW 35116, and three lines from the 51st ESWYT, carrying two- or three-gene combinations remained immune at both seedling and adult-plant stages across all three years. These genotypes constitute valuable genetic resources for pyramiding durable APR genes and accelerating the development of stripe rust-resistant wheat cultivars.

## 1. Introduction

Wheat (*Triticum aestivum* L.) is one of the most important cereal crops worldwide, providing nearly 20% of the calories and protein consumed by humans and serving as a staple food for approximately one-third of the global population [1]. Globally, wheat is cultivated on about 225 million ha with an annual production exceeding 750 million tonnes, while India is the second-largest producer, contributing nearly 13% of global production [2]. As bread wheat occupies nearly 95% of the cultivated wheat area worldwide, sustaining its productivity is essential for global food and nutritional security [3]. However, achieving higher and stable wheat production remains challenging because of several biotic constraints, among which stripe (yellow) rust, caused by *Puccinia striiformis* f. sp. *tritici* (Pst), is one of the most destructive diseases. Stripe rust occurs predominantly in cool and humid wheat-growing regions of North America, Europe, North Africa, South Asia, and Central and West Asia, where wind-borne urediniospores facilitate rapid long-distance dissemination [4]. Globally, the disease causes an estimated annual yield loss of approximately 5.47 million tonnes and continues to threaten wheat production through recurrent epidemics [5]. Recent reports indicate that infection by Pst can result in yield reductions ranging from 20% to 40% and in severe cases, losses may reach up to 100% in susceptible cultivars [6]. In India, stripe rust is a recurring constraint in the Northern Plains and Himalayan foothills, affecting nearly 10 million ha annually and causing yield losses ranging from 4% to complete crop failure under favourable environmental conditions [7]. Infection reduces photosynthetic efficiency, assimilate translocation, grain filling, and thousand-grain weight, resulting in substantial yield reductions, particularly in susceptible cultivars [8,9].

The management of stripe rust is further complicated by the rapid evolution of new virulent pathotypes capable of overcoming resistance deployed in widely cultivated wheat cultivars. In India, recently evolved pathotypes, including 46S119, 110S119, 110S84, and 238S119, have rendered important resistance genes such as *Yr9* and *Yr27* ineffective in popular cultivars including PBW 343 and HD 2967 [10]. Consequently, identifying new and durable sources of resistance has become a major priority for wheat improvement programmes.

More than 87 officially designated stripe rust resistance (*Yr*) genes, approximately 100 temporarily designated genes, and over 360 quantitative trait loci (QTL) have been reported in wheat [11]. These resistance loci are broadly categorized into all-stage resistance (ASR) and adult plant resistance (APR). ASR is generally governed by major race-specific genes that provide complete resistance at the seedling stage but are frequently overcome by newly emerging pathogen races [12]. In contrast, APR is typically race-non-specific, quantitatively inherited, and expressed after the seedling stage, providing partial yet durable resistance across diverse environments [13]. Consequently, APR has become the cornerstone of breeding strategies aimed at achieving durable stripe rust resistance. Among the characterized APR genes, *Yr18*/*Lr34*/*Sr57*/*Pm38*, *Yr29*/*Lr46*/*Sr58*/*Pm39*, and *Yr46*/*Lr67*/*Sr55*/*Pm46* are regarded as the most effective sources of durable, broad-spectrum resistance. These genes confer partial resistance not only to stripe rust but also to leaf rust, stem rust, and powdery mildew [14,15]. Importantly, their effects are largely additive, and cultivars carrying two or more APR genes generally exhibit substantially higher and more durable field resistance than those possessing individual genes, making them ideal targets for marker-assisted gene pyramiding [16,17].

Despite considerable advances in understanding stripe rust resistance, important gaps remain. Most previous studies have evaluated either seedling resistance or adult plant resistance independently, whereas relatively few have integrated phenotypic screening at both developmental stages with molecular characterization of major APR genes in a diverse germplasm panel. Furthermore, the frequency, distribution, and pyramided combinations of *Yr18*, *Yr29*, and *Yr46* have not been comprehensively investigated in indigenous and exotic wheat germplasm under the pressure of the currently predominant Indian stripe rust pathotypes. As a result, the contribution of APR gene combinations to durable field resistance under contemporary Indian pathogen populations remains insufficiently understood. The novelty of the present study lies in the integrated evaluation of a diverse panel of 200 indigenous and exotic wheat genotypes using both phenotypic and molecular approaches. The genotypes were screened against the five predominant Indian stripe rust pathotypes (46S119, 110S119, 238S119, 110S84, and 47S103) at both seedling and adult plant stages, followed by molecular postulation of the major APR genes *Yr18*, *Yr29*, and *Yr46* using closely linked SSR markers. This comprehensive approach enabled simultaneous assessment of resistance phenotypes, determination of APR gene frequencies and pyramided combinations, and evaluation of their contribution to durable slow-rusting resistance under the prevailing Indian pathogen population. We hypothesized that wheat genotypes carrying pyramided combinations of *Yr18*, *Yr29*, and *Yr46* would exhibit significantly greater and more durable resistance to the predominant Indian stripe rust pathotypes than genotypes carrying individual APR genes or lacking these loci. We further hypothesized that integrating multi-stage phenotypic evaluation with marker-assisted postulation of APR genes would facilitate the identification of previously unrecognized donor genotypes possessing durable resistance suitable for wheat improvement programmes. Therefore, the objectives of this study were to (i) evaluate 200 indigenous and exotic wheat genotypes for resistance to the predominant Indian *P. striiformis* pathotypes at both seedling and adult plant stages; (ii) postulate the presence of the APR genes*Yr18*, *Yr29*, and *Yr46* using gene-linked SSR markers; (iii) determine the frequency and pyramided combinations of these APR genes; (iv) examine the relationship between APR gene combinations and slow-rusting resistance; and (v) identify superior donor genotypes for marker-assisted breeding of durable stripe rust-resistant wheat cultivars.

## 2. Results

### 2.1. Variation in Stripe Rust Resistance Components Under Field Conditions

**Figure 1:**
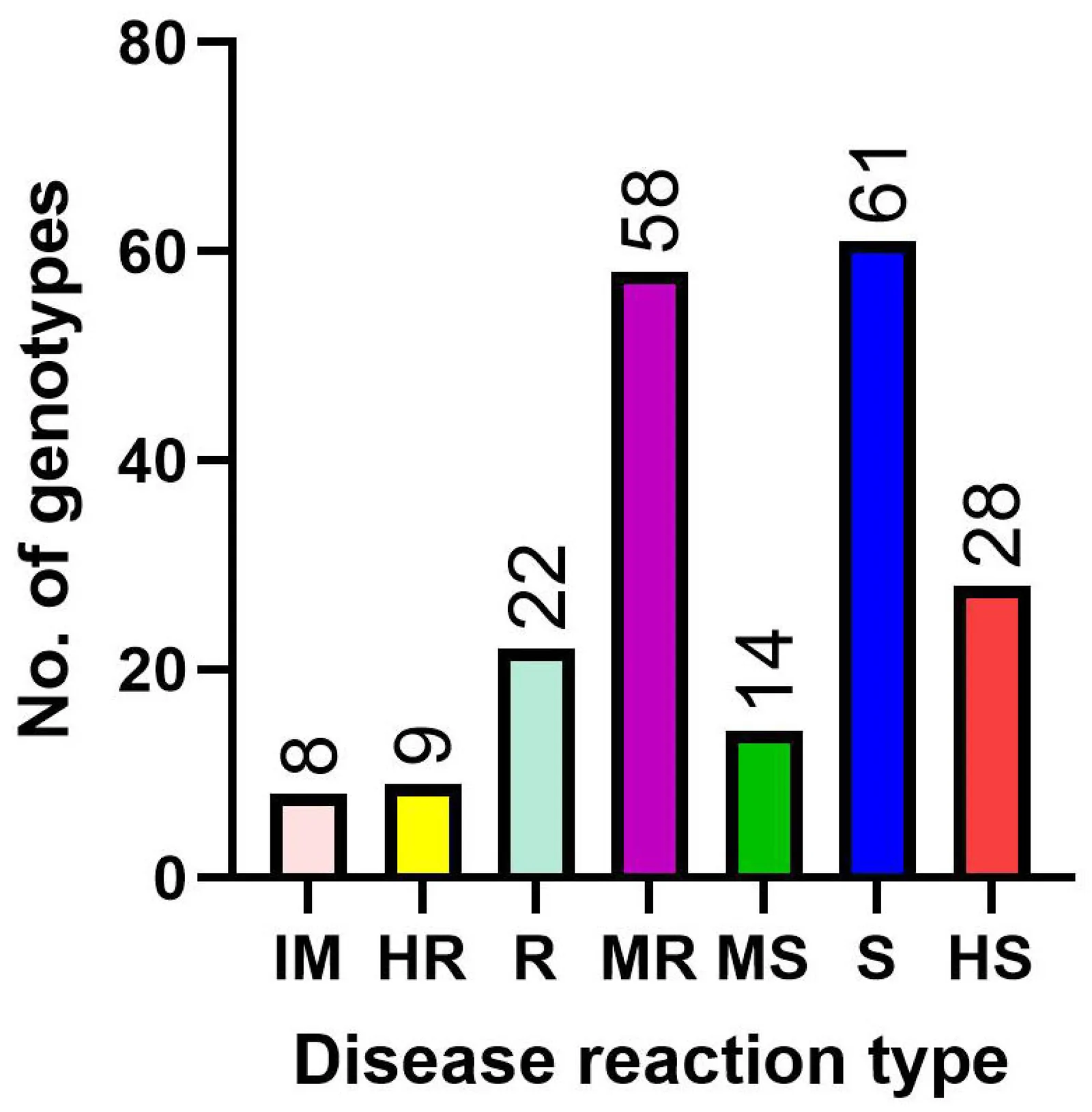
Distribution of the 200 tested genotypes among final disease severity reaction classes against stripe rust, 2021–2024. Immune (IM), highly resistant (HR), resistant (R), moderately resistant (MR), moderately susceptible (MS), susceptible (S) and highly susceptible (HS).

**Figure 2:**
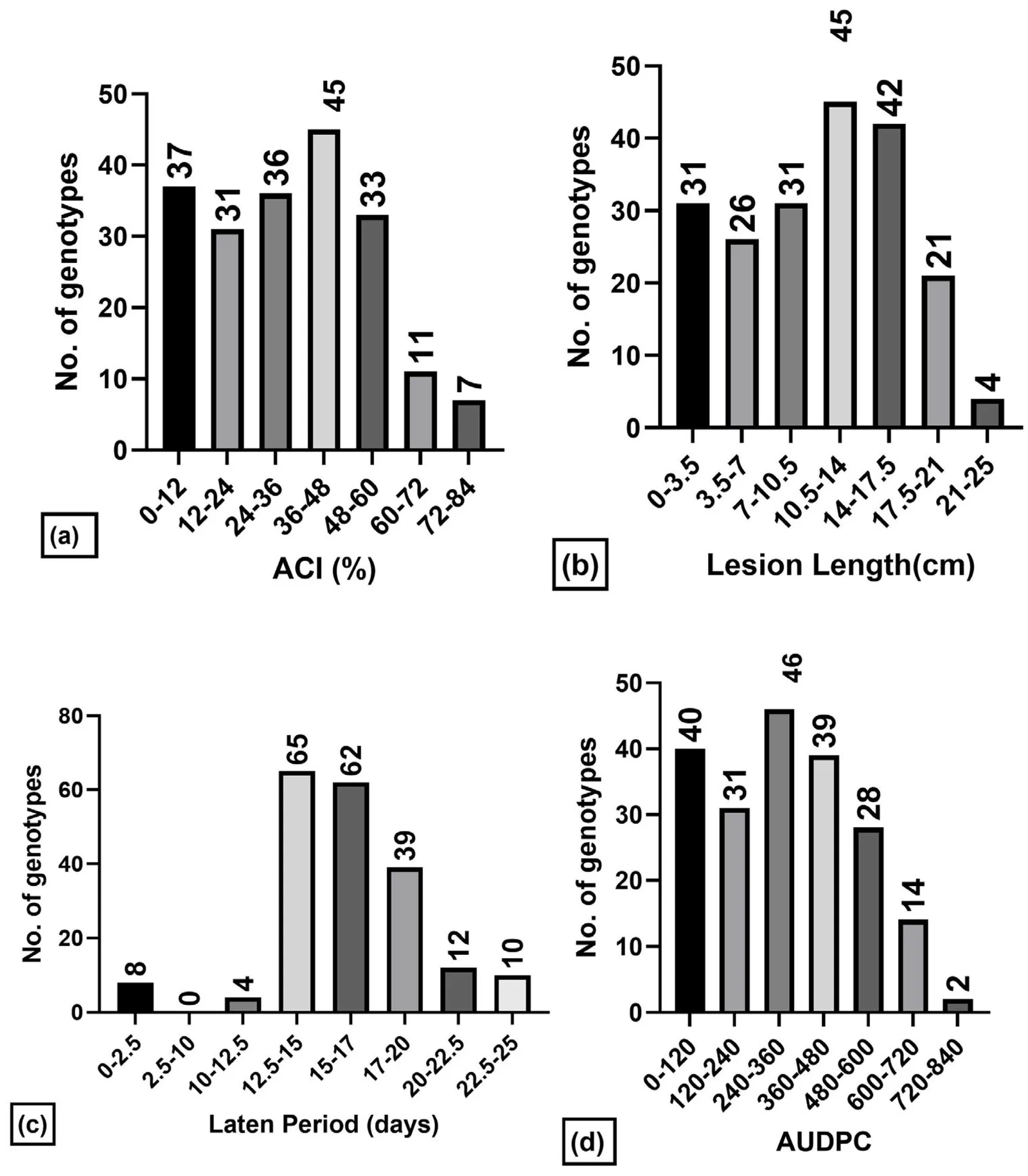
Distribution of the 200 wheat genotypes for stripe rust resistance components. (**a**) Average coefficient of infection (ACI); (**b**) lesion length; (**c**) latent period; (**d**) area under the disease progress curve (AUDPC).

Significant differences (*p* < 0.05) were detected among the 200 genotypes for all disease-resistance components (latent period, lesion length, FDS, ACI and AUDPC) recorded over the three field seasons (2021–2024) (Table 1), confirming substantial genetic variability for slow-rusting resistance in the panel. Based on the phenotyping results, distinct and consistent reactions against stripe rust disease were found; eight genotypes showed a consistently immune response across the years (Figure 1). The latent period was longest (20–25 days) in 22 genotypes, most commonly fell within 12.5–17.5 days (127 genotypes), and was shortest (10–12.5 days) in only 4 genotypes (Figure 2). Based on ACI, 37 genotypes showed values below 12% (resistant to highly immune), 45 genotypes fell between 36 and 48%, and 51 genotypes exceeded 50% (moderately to highly susceptible) (Figure 2). AUDPC values were lowest (0–120) in 40 genotypes, indicating high resistance, and highest (600–840) in only 16 genotypes (Figure 2). For lesion length, 31 genotypes fell in the 0–3.5 cm range (highly resistant to immune), while 112 genotypes exceeded 10.5 cm (highly susceptible) (Figure 2), together indicating that the majority of susceptibility was concentrated in a subset of genotypes, while a meaningful proportion showed strong slow-rusting characteristics.

Field reactions recorded consistently across all three years (Table 1) classified eight genotypes (BW 35109, BW 35110, BW 35112, BW 35114, BW 35116, 51st ESWYT 145, 51st ESWYT 146 and 51st ESWYT 147) as immune, while 9, 22, 58, 14, 61 and 28 genotypes were highly resistant, resistant, moderately resistant, moderately susceptible, susceptible and highly susceptible, respectively. In percentage terms, 4% of genotypes were immune, 4.5% highly resistant, 11% resistant, 29% moderately resistant, 7% moderately susceptible, 30.5% susceptible and 14% highly susceptible. High and uniform disease pressure was confirmed each year by final disease severity of 90% recorded on all susceptible checks (Agra Local, Kharchia 65, PBW 343 and A-9-30-1).

### 2.2. Seedling Reactions to Five Stripe Rust Pathotypes

Seedling infection types recorded for the 200 genotypes and four susceptible checks against the five predominant pathotypes revealed marked pathotype-specific variation in resistance frequency (Table 2). Against pathotype 46S119, 33% of genotypes were resistant, 2.5% moderately resistant, 15% moderately susceptible and 49.5% susceptible. Resistance frequency was lower against 110S119 (12% resistant) and 238S119 (16% resistant), for which the majority of genotypes (71.5% and 75%, respectively) were susceptible. In contrast, resistance was more frequent against 110S84 (34% resistant) and 47S103 (33.5% resistant, with a further 8.5% moderately susceptible only, and no fully susceptible class recorded), indicating that a subset of genotypes retained race-specific seedling resistance to the more recently evolved pathotypes.

### 2.3. Relationships Among Stripe Rust Resistance Components

**Figure 3:**
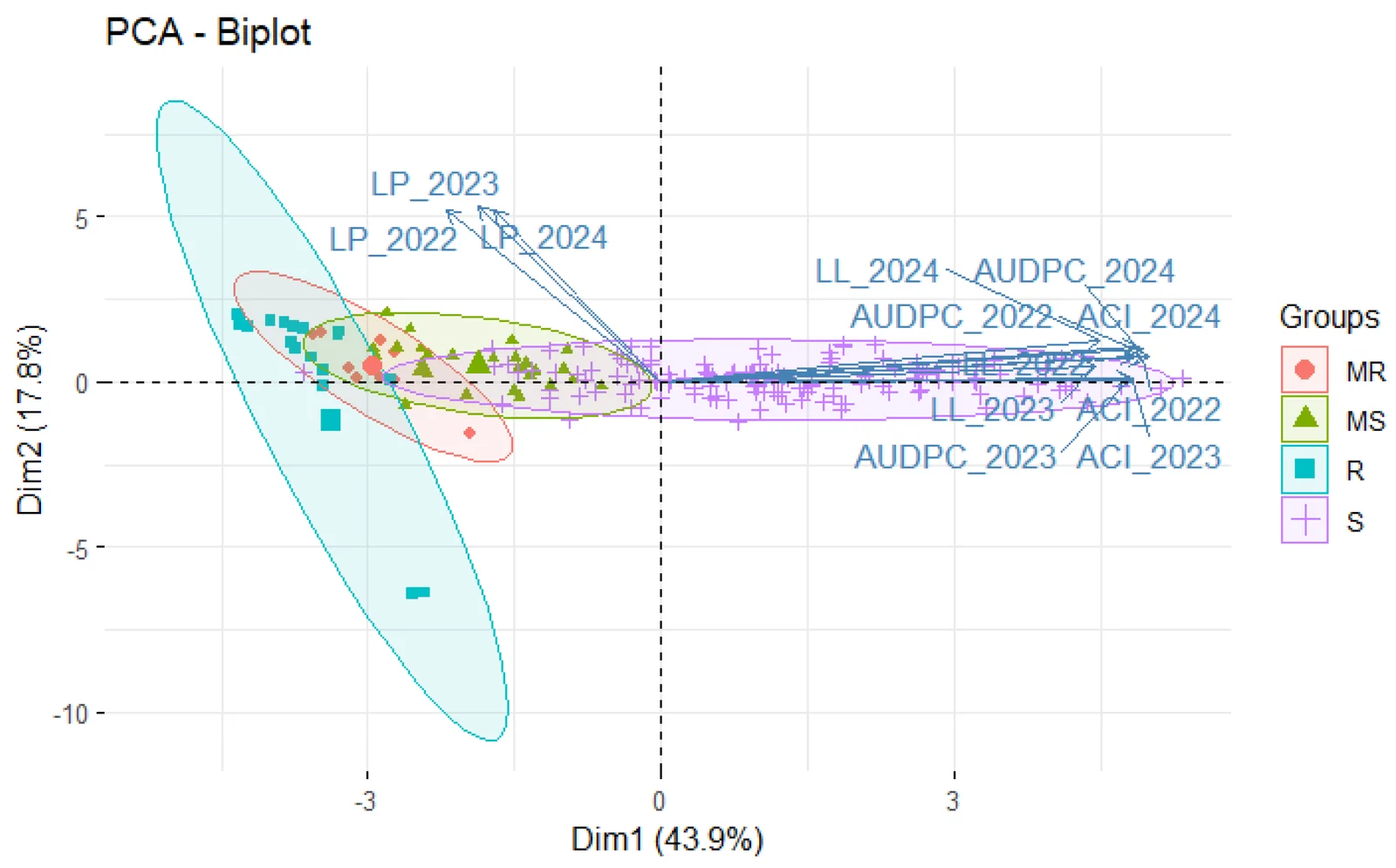
Principal component analysis (PCA) biplot of 200 bread wheat genotypes based on stripe rust disease-related traits (lesion length, ACI, AUDPC, latent period).

Principal component analysis of the disease-related traits (lesion length, ACI, AUDPC, latent period) separated genotypes into four groupings corresponding to resistant (R), moderately resistant (MR), moderately susceptible (MS) and susceptible (S) reaction classes (Figure 3). The first two principal components explained 43.9% and 17.8% of total variance, respectively. Resistant genotypes clustered together, characterized by longer latent periods and lower AUDPC and ACI values across seasons, indicating slower disease progression and reduced infection; moderately resistant genotypes occupied an intermediate position, while susceptible and moderately susceptible genotypes clustered separately, associated with higher AUDPC and ACI and more rapid disease onset, particularly in 2022–2024.

### 2.4. Molecular Postulation and Distribution of Adult Plant Resistance Genes

**Figure 4:**
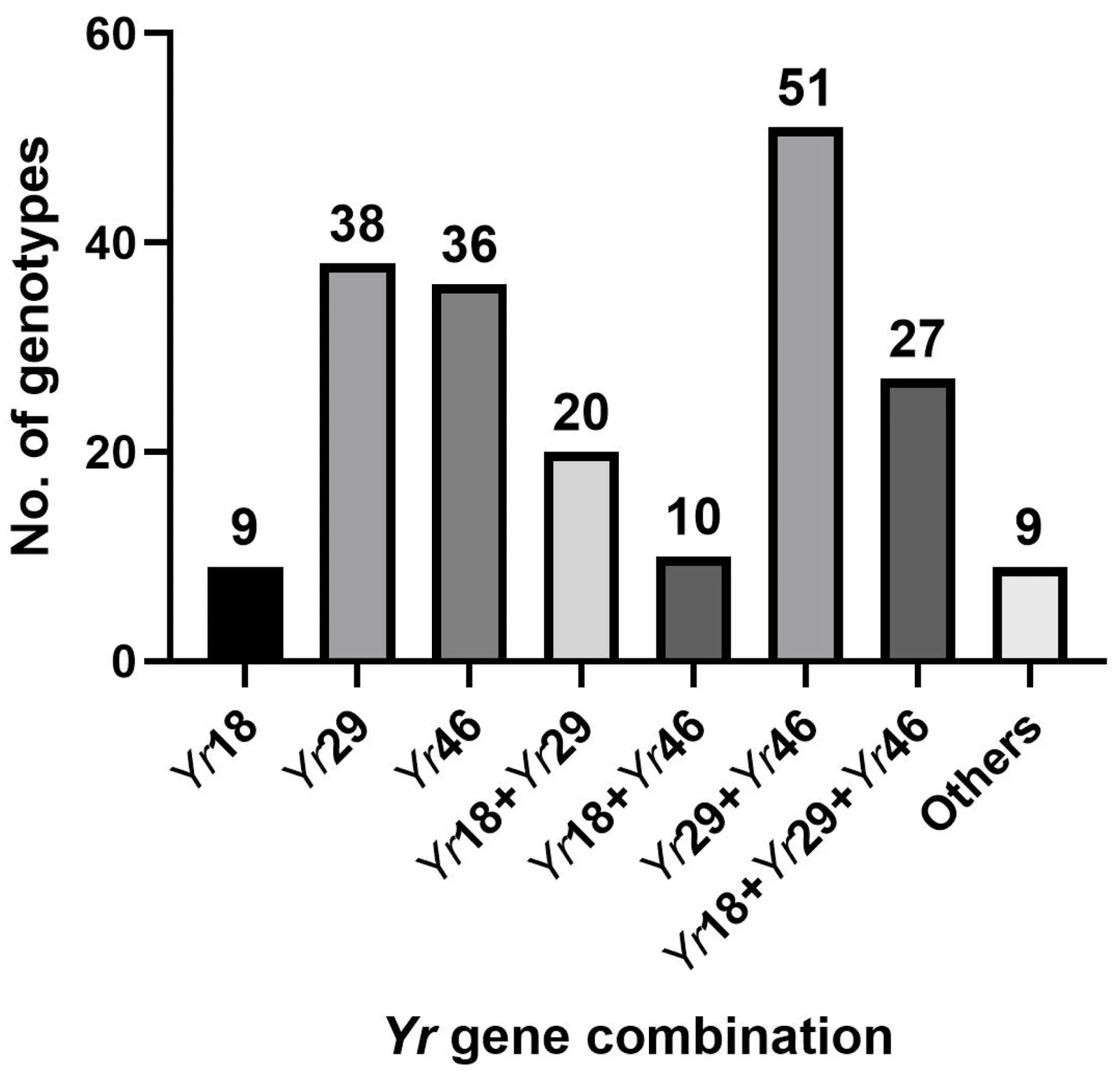
Distribution of *Yr18*, *Yr29* and *Yr46* adult plant resistance gene combinations among the 200 wheat genotypes.

Molecular marker analysis of all 200 genotypes detected specific amplicons corresponding to *Yr18* (marker *csLV34*), *Yr29* (*wmc44*) and *Yr46* (*gwm192*) in both the positive controls and the test panel (Figures S1–S3). Considered individually, *Yr18*, *Yr29* and *Yr46* were present in 9, 38 and 36 genotypes, respectively; two-gene combinations were detected in 81 genotypes (*Yr18*+*Yr29*, 20; *Yr18*+*Yr46*, 10; *Yr29*+*Yr46*, 51), and the three-gene combination *Yr18*+*Yr29*+*Yr46* was present in 27 genotypes (Figure 4). Across all genotypes, *Yr29* was the most frequently detected gene, present in around 60% of the panel, either alone or in combination, while *Yr46* was detected in around 57% of genotypes (Figure 4). Nine genotypes carried none of the three markers, suggesting that resistance in these lines may be conferred by additional, unidentified genes.

### 2.5. Association Between Number of APR Genes and Disease Progression Traits

**Figure 5:**
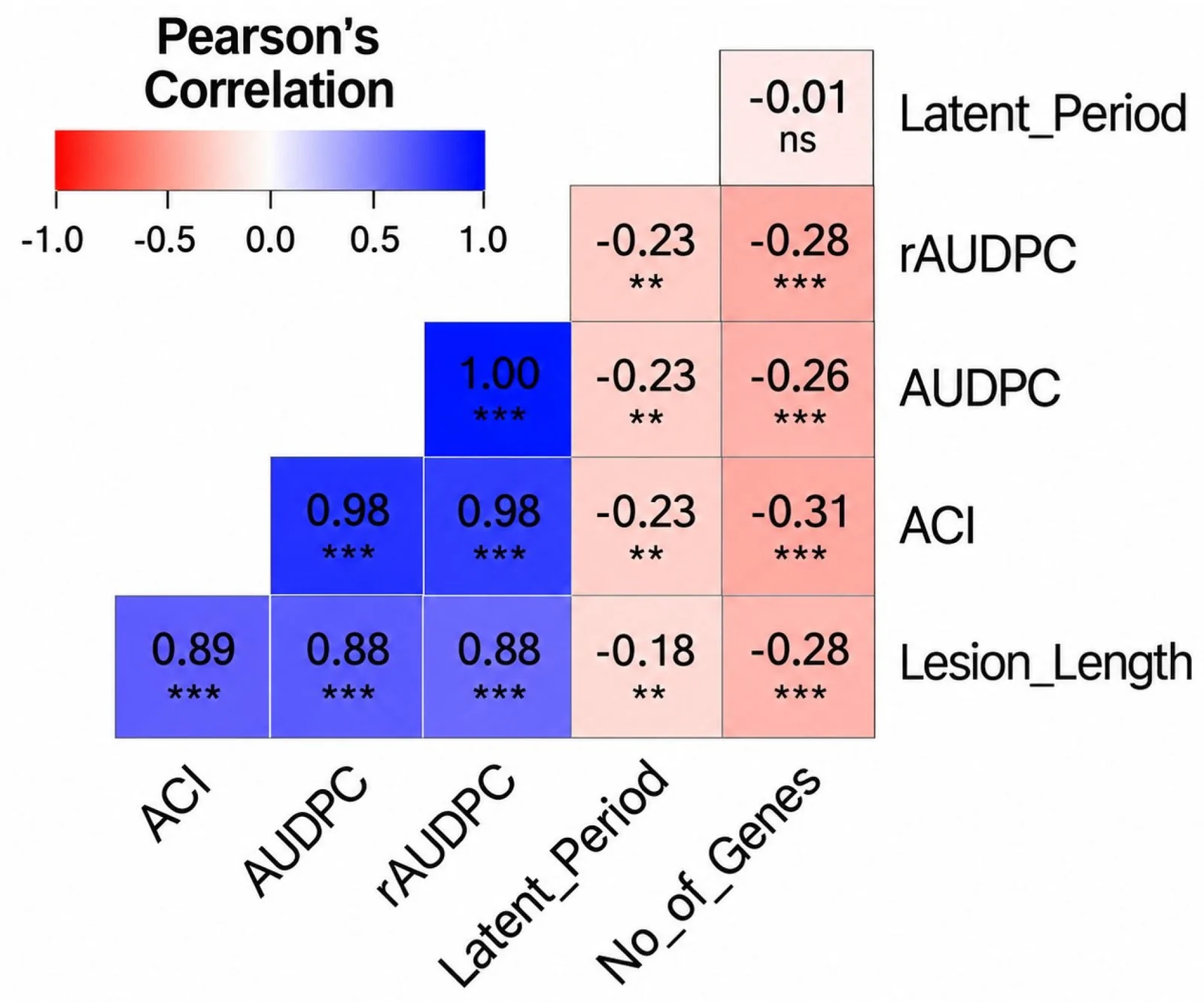
Pearson correlation-based relationships among stripe rust disease progression traits and the number of *Yr* resistance genes present in the genotypes. ns: non-significant; **: *p* < 0.05; ***: *p* ≤ 0.01.

**Figure 6:**
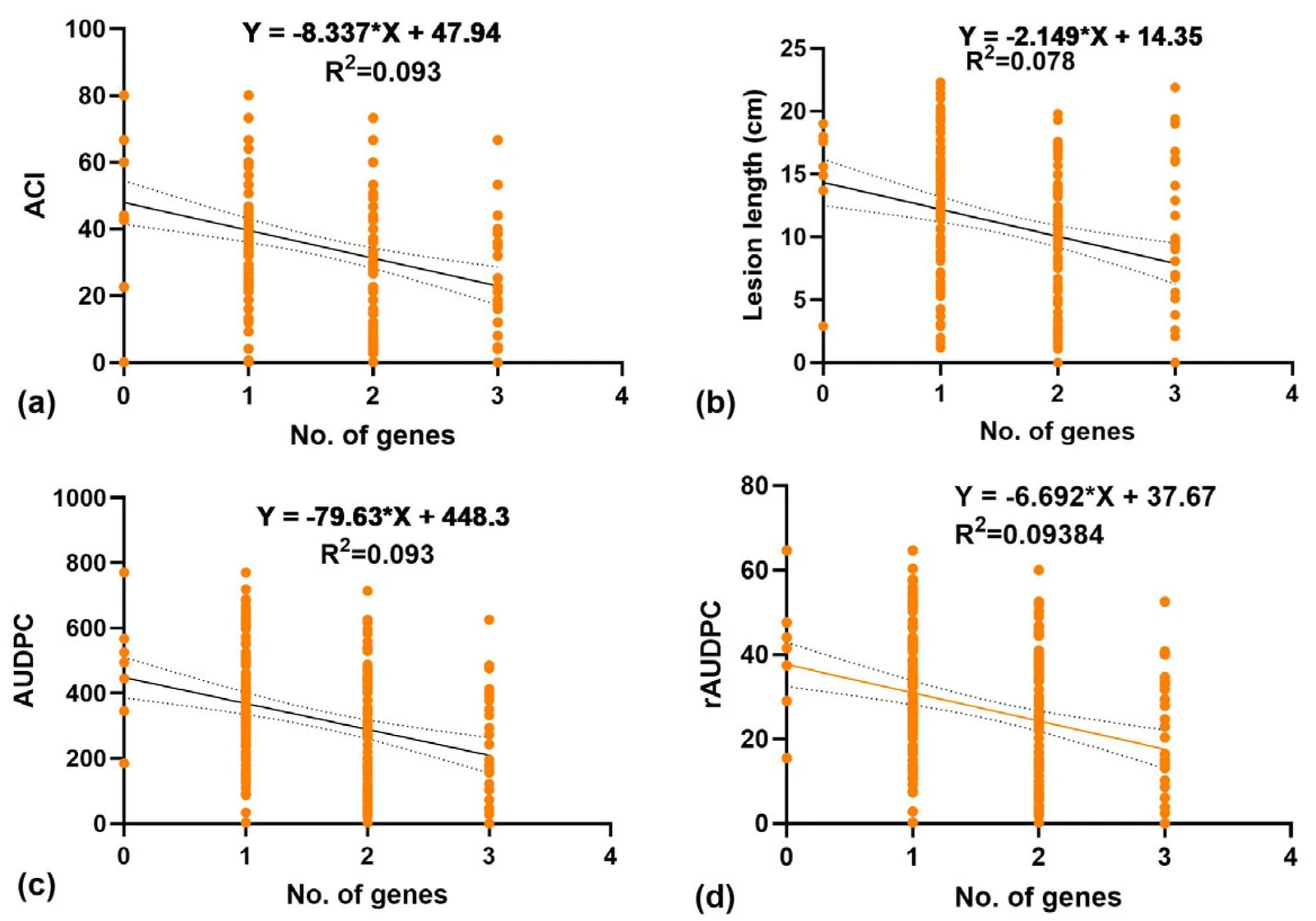
Relationship between the number of pyramided adult plant resistance genes and stripe rust progression traits: (**a**) average coefficient of infection (ACI); (**b**) lesion length; (**c**) AUDPC; (**d**) relative AUDPC (rAUDPC).

Pearson correlation analysis among stripe rust progression traits and the number of pyramided *Yr* genes (Figure 5) showed AUDPC and rAUDPC to be perfectly correlated (r = 1.00, *p* < 0.001) and strongly associated with ACI (r = 0.98, *p* < 0.001) and lesion length (r = 0.88, *p* < 0.001). All three traits (AUDPC, rAUDPC, ACI) were negatively correlated with the number of APR genes present (r = −0.28 to −0.31, *p* < 0.001). Latent period showed weak-to-moderate negative correlations with AUDPC, rAUDPC and ACI (r = −0.23, *p* < 0.001 for each) but was not significantly correlated with gene number (r = −0.01, ns). Consistent with these correlations, mean ACI declined from approximately 50% in genotypes carrying a single APR gene to approximately 30% in genotypes carrying all three genes, and AUDPC, rAUDPC and lesion length showed similar declining trends with increasing gene number (Figure 6), confirming an additive contribution of pyramided APR genes to reduced disease progression.

## 3. Discussion

Stripe rust, caused by *P. striiformis* f. sp. *tritici* (Pst), continues to be one of the most destructive diseases of wheat worldwide because of the remarkable evolutionary capacity of the pathogen to generate new virulent pathotypes that rapidly overcome race-specific resistance genes deployed in commercial cultivars. The frequent breakdown of major *Yr* genes has shifted breeding strategies from reliance on all-stage resistance (ASR) towards the deployment of durable adult plant resistance (APR), particularly through pyramiding multiple slow-rusting genes. Consequently, the identification of novel APR donors possessing broad-spectrum and environmentally stable resistance has become a major objective of wheat improvement programmes worldwide. In the present investigation, three years of field phenotyping, seedling evaluation against five predominant Indian Pst pathotypes, epidemiological characterization, multivariate statistical analysis, and molecular marker validation were integrated to comprehensively characterize 200 indigenous and exotic wheat genotypes for stripe rust resistance. The results demonstrated extensive diversity for both qualitative and quantitative resistance, identified highly resistant donor genotypes, and highlighted the importance of APR gene pyramiding for achieving durable resistance.

A prerequisite for durable resistance breeding is the availability of genetically diverse resistance sources. The significant variation observed among the evaluated genotypes for latent period (LP), lesion length (LL), final disease severity (FDS), average coefficient of infection (ACI), and area under the disease progress curve (AUDPC) clearly demonstrated the existence of substantial quantitative genetic variation governing stripe rust resistance. Unlike major race-specific resistance genes, quantitative resistance is conditioned by several genes with minor additive effects that collectively restrict pathogen growth and reduce epidemic development, thereby providing greater durability under diverse environments [21,22]. Similar conclusions were reached by Pathan and Park [23,24], who demonstrated that epidemiological parameters such as AUDPC and ACI are reliable indicators of slow-rusting resistance, while Prasad et al. [25] and Madahana et al. [26] further confirmed their effectiveness for identifying durable resistance across multiple environments. The present findings therefore reinforce the concept that breeding for durable resistance should rely on the combined assessment of several epidemiological traits rather than disease severity alone.

Among the epidemiological components, latent period emerged as one of the most informative indicators of slow-rusting resistance. Extension of the latent period delays urediniospore production, reduces the number of pathogen generations during the cropping season, and consequently suppresses epidemic development under field conditions [27]. Previous studies have shown that slow-rusting cultivars infected with *P. striiformis* produce fewer uredinia, smaller pustules, and lower urediniospore production than susceptible cultivars [21,28]. Consistent with these observations, twenty-two wheat genotypes in the present study exhibited latent periods exceeding 20 days together with significantly lower disease severity, reduced lesion development, and lower AUDPC values, demonstrating an integrated expression of quantitative resistance. Similar variability has been reported by Ghannadha et al. [29] Dehghani et al. [30], and Singh et al. [31], suggesting that latent period is under both additive and dominant genetic control and represents a valuable selection criterion for breeding durable resistance.

Lesion length also contributed significantly to the expression of quantitative resistance. Considerable variation was observed among the germplasm, ranging from complete absence of lesions to lesions exceeding 22 cm. Eight genotypes consistently remained lesion-free, whereas several additional entries developed only minute lesions despite continuous disease pressure, indicating efficient restriction of pathogen colonization following infection. Earlier studies similarly reported that reduced lesion size, together with prolonged latent period and reduced sporulation, constitutes one of the characteristic features of slow-rusting resistance [32,33,34]. The present findings therefore demonstrate that lesion development is an important phenotypic marker for identifying quantitatively resistant germplasm and should be incorporated into routine field screening programmes.

Field evaluation under artificial epiphytotic conditions further demonstrated that durable resistance is best characterized through the integrated expression of multiple epidemiological components. Resistant genotypes consistently exhibited lower FDS, ACI, AUDPC, and lesion length together with prolonged latent periods, whereas susceptible entries showed rapid disease progression characterized by early sporulation and severe epidemic development. Among these parameters, AUDPC proved particularly valuable because it integrates disease intensity throughout the crop cycle and therefore provides a comprehensive measure of epidemic development. Similar observations have been reported by several workers [23,35], who recognized AUDPC as one of the most reliable indicators of adult plant resistance. Furthermore, the strong positive correlations observed among disease severity, lesion length, ACI, and AUDPC indicate that these epidemiological parameters represent interconnected stages of pathogen development and collectively provide a robust phenotypic framework for selecting durable resistance in breeding populations. The multivariate analysis further validated these observations by revealing the underlying structure of phenotypic variation. Principal component analysis showed that the first two principal components explained the majority of the observed variation, with disease progression parameters contributing most strongly to genotype discrimination. Resistant genotypes clustered with prolonged latent periods and lower AUDPC and ACI values, whereas susceptible entries grouped according to rapid disease progression and high terminal disease severity. These findings demonstrate the usefulness of multivariate statistical approaches for simultaneously evaluating multiple resistance components, reducing redundancy among correlated traits, and identifying genetically diverse parents possessing complementary resistance mechanisms.

The multi-year field evaluation under uniform artificial epiphytotic conditions enabled the identification of highly stable resistance sources. Eight wheat genotypes (BW 35109, BW 35110, BW 35112, BW 35114, BW 35116, 51st ESWYT 145, 51st ESWYT 146, and 51st SWYT 147) consistently expressed immune reactions throughout three consecutive cropping seasons, whereas susceptible checks developed nearly 90% disease severity. In addition, twenty-two genotypes exhibiting prolonged latent periods together with lower AUDPC and disease severity represented valuable sources of slow-rusting resistance. The stability of their resistance across years strongly suggests the presence of environmentally stable APR mechanisms. Approximately one-third of the evaluated germplasm displayed moderate to high levels of resistance, whereas only a limited proportion remained highly susceptible, indicating the availability of substantial genetic diversity that can be exploited to broaden the resistance base of future wheat cultivars. It is also worth noting that the eight genotypes classified as immune recorded a latent period, lesion length, ACI and AUDPC of exactly zero in every season, whereas adult plant resistance is typically expressed as slow-rusting rather than absolute immunity. This pattern raises the possibility that these lines, in addition to carrying two- or three-gene APR combinations, may also possess all-stage resistance (ASR) genes effective against the Pst pathotypes.

Evaluation of seedlings against five predominant Indian pathotypes (46S119, 110S119, 238S119, 110S84, and 47S103) revealed considerable pathotype-specific variation in resistance frequency, reflecting the continuously evolving virulence spectrum of Indian Pst populations. Resistance frequencies ranged from only 12–16% against pathotypes 110S119 and 238S119 to nearly one-third of the germplasm against pathotypes 46S119, 110S84, and 47S103. These findings agree with recent reports documenting the emergence of new virulence combinations capable of overcoming widely deployed resistance genes such as *Yr9*, *Yr17*, *Yr27*, *Yr43*, *Yr44*, and *Yr76* [9]. Importantly, several genotypes that were susceptible at the seedling stage expressed high levels of resistance under field conditions, confirming the presence of developmentally regulated APR genes. The seedling assays were included to characterise race-specific, all-stage resistance (ASR) independently of the three APR genes under study, since *Yr18, Yr29* and *Yr46* are not expected to condition the seedling response; the seedling and adult-plant datasets are therefore complementary rather than directly comparable measures of the same resistance mechanism, and seedling infection type was not used to validate or contradict the SSR-based APR gene calls. Such contrasting responses between seedling and adult plant stages reinforce the importance of evaluating breeding materials across different growth stages and combining race-specific ASR genes with durable APR genes for sustainable stripe rust management.

Molecular marker analysis demonstrated that the pleiotropic APR genes *Yr18*, *Yr29*, and *Yr46* are widely distributed within the evaluated germplasm, although their frequencies differed substantially. *Yr29* was the most prevalent gene, occurring in approximately 68% of the genotypes, followed by *Yr46*, whereas *Yr18* occurred less frequently but was commonly detected in pyramided combinations. These observations corroborate previous reports describing the widespread deployment of *Yr29*/*Lr46* in CIMMYT-derived germplasm and its important contribution to durable resistance against multiple rust diseases [36,37]. Likewise, *Yr18*/*Lr34* remains one of the best-characterized pleiotropic APR genes because of its ATP-binding cassette transporter-mediated resistance against stripe rust, leaf rust, stem rust, and powdery mildew [38]. Similar distributions of these genes have been reported from Australia, China, Ethiopia, India, Nepal, Pakistan, and Europe [39,40,41,42], indicating that they continue to constitute the foundation of durable rust resistance breeding worldwide. In contrast, Majeed et al. [43] reported that *Yr46* was detected in only 5.3% of the Pakistani wheat cultivars evaluated, with its occurrence largely restricted to cultivars released before 1965, except for Tatara and Khatakwa. The authors highlighted the limited deployment of *Yr46* in modern Pakistani wheat cultivars and the significant allelic effect of *Yr46* in relation to the reduction of stripe rust severity by 20–36.2% at the adult plant stage. It is also important to consider the nature and limitations of the molecular markers used for Yr gene postulation in the present study. The SSR markers wmc44 and gwm192 are linked to Yr29 and Yr46, respectively, and are located in flanking genomic regions rather than within the corresponding resistance genes. Thus, detection of the expected marker alleles provides supportive evidence for the presence of these resistance loci; however, recombination between the marker and the causal gene cannot be completely excluded. Such recombination could account for a small proportion of discordant marker–phenotype combinations and represents an inherent limitation of SSR-based gene postulation using linked markers. In contrast, *csLV34* is a gene-derived STS marker associated with *Yr18* and is therefore less susceptible to the specific limitation arising from recombination between a linked marker and the target locus. Accordingly, the *Yr29* and Yr46 assignments based on wmc44 and gwm192, respectively, should be interpreted as marker-based postulations rather than definitive confirmation of the corresponding resistance genes. Future studies should validate these postulations using gene-specific sequence-based markers or high-throughput KASP SNP assays, which would provide more direct and reliable evidence for the presence of the underlying resistance loci. Such validation would also strengthen the interpretation of marker–phenotype associations and facilitate more precise characterization and deployment of APR gene combinations in wheat breeding programmes.

One of the most important outcomes of the present study was the clear demonstration of the additive advantage conferred by APR gene pyramiding. More than half of the evaluated germplasm carried combinations of two or more APR genes, and all eight immune genotypes possessed either two- or three-gene combinations. Disease progression declined progressively with increasing numbers of APR genes, resulting in significant reductions in ACI, AUDPC, lesion length, and terminal disease severity. Genotypes carrying the *Yr18*+*Yr29*+*Yr46* combination consistently exhibited substantially greater resistance than those possessing individual genes, confirming the cumulative and complementary effects of APR gene pyramiding. Similar synergistic interactions among slow-rusting genes have been highlighted by several workers [15,16,17,44,45], who demonstrated that combining multiple APR genes can produce near-immune levels of resistance while enhancing long-term durability by reducing the probability of pathogen adaptation. These findings strongly support the strategic deployment of multiple APR genes as the cornerstone of sustainable stripe rust resistance breeding. Interestingly, several highly resistant genotypes lacked diagnostic markers for *Yr18*, *Yr29*, and *Yr46*, yet consistently expressed excellent field resistance across years. This observation strongly suggests the presence of additional APR genes or previously unidentified quantitative resistance loci contributing to durable resistance. Similar unexplained resistance has been reported in Chinese and Ethiopian wheat germplasm [46,47], indicating that considerable untapped genetic variation remains available within global wheat collections. These genotypes therefore represent valuable resources for quantitative trait locus (QTL) mapping, genome-wide association studies, genomic prediction, and resistance gene discovery.

## 4. Conclusions

The present study demonstrates that integrating long-term field phenotyping with epidemiological analyses, pathotype-specific screening, multivariate statistics, and molecular marker validation provides a comprehensive framework for identifying durable stripe rust resistance. The identification of highly resistant donor genotypes carrying multiple APR genes, together with resistant accessions lacking known APR markers, considerably broadens the genetic resources available for stripe rust improvement. These findings provide a strong foundation for marker-assisted selection, genomic-assisted breeding, and resistance gene discovery, ultimately facilitating the development of climate-resilient wheat cultivars with broad-spectrum and durable resistance against the rapidly evolving populations of *P. striiformis* f. sp. *tritici*.

## 5. Materials and Methods

### 5.1. Plant Material and Stripe Rust Pathotypes

A panel of 200 wheat (*Triticum aestivum* L.) germplasm accessions of diverse genetic backgrounds, obtained from the Germplasm Resource Unit, ICAR-Indian Institute of Wheat and Barley Research (IIWBR), Karnal, Haryana, India, was used in this study (genotype pedigrees are listed in Table S1). Five predominant Indian pathotypes of *Puccinia striiformis* f. sp.*tritici*, namely 238S119, 110S119, 110S84, 46S119 and 47S103 [5,10], were used to characterize stripe rust reaction of the panel at the seedling and adult-plant stages. The susceptible cultivars Agra Local, Kharchia 65, PBW 343 and A-9-30-1/HD 2967 were included as susceptible checks.

### 5.2. Seedling Resistance Test Under Glasshouse Conditions

Seedling reaction was assessed separately against each of the five pathotypes. Genotypes, along with the susceptible checks, were sown in aluminium trays (29 × 12 × 7 cm) containing sterilized soil and maintained in an insect-proof glasshouse. Each tray contained 18 wheat genotypes along with a susceptible check, and 5–6 seeds of each genotype were planted in separate hills. Seven- to eight-day-old seedlings at the first-leaf stage were inoculated with a fresh urediniospore suspension of the respective pathotype [spores mixed in light mineral oil, Soltrol^®^ (Chevron Phillips Chemical, The Woodlands, TX, USA)] using a hand atomizer, and incubated for 24 h in a humidity/dew chamber at 10 ± 2 °C in darkness to promote infection, before being transferred to the glasshouse at 15–20 °C with supplementary lighting. Infection type (IT) was scored 14–16 days after inoculation on the 0–4 scale of Stakman et al. [18], as modified by Nayar et al. [19] and Bhardwaj et al. [20] (Table S2), and reactions were grouped into resistant (R), moderately resistant (MR), moderately susceptible (MS) and susceptible (S) classes. Experiments were repeated twice.

### 5.3. Evaluation of Adult Plant Resistance Under Field Conditions

The same 200 genotypes were evaluated for APR over three consecutive crop seasons (2021–2024) at the Crop Protection field, ICAR-IIWBR, Karnal, India. Following field preparation, approximately 30–35 seeds of each genotype were sown in two 1.5 m rows spaced 22.5 cm apart, with irrigation and fertilizer application following standard agronomic practice. The field experiments followed an augmented block design, with 200 test genotypes distributed across ten blocks, comprising approximately 20 unreplicated test genotypes per block. A susceptible check was replicated in each block and served as a common reference for comparison among blocks. Every 20th row was sown with a mixture of susceptible check cultivars to serve as a spreader/infector row, and a perpendicular spreader row of susceptible checks bordered the plot. Artificial epiphytotic conditions were created by spraying infector rows with a urediniospore suspension of the mixed prevalent pathotypes (238S119, 110S119, 110S84, 46S119 and 47S103) in water containing 0.01% Tween-20, applied in the evening with a hand sprayer three times at 10-day intervals beginning 65 days after sowing; infected leaves were additionally rubbed onto healthy seedlings to promote uniform disease build-up. Rust spores generated in the spreader rows were periodically harvested and reapplied to the infector rows to maintain uniform, high inoculum pressure across the plot.

### 5.4. Disease Scoring and Resistance Components

Host response and disease severity were recorded on the two-row plot of each genotype at 7-day intervals, five times per season, from February to March in each of the three years, using the modified Cobb scale [48] once disease severity in the susceptible checks reached approximately 40%. Adult plant infection type was classified as susceptible (S), moderately susceptible (MS), moderately resistant (MR), resistant (R) or immune (I), following Roelfs et al. [27]. Latent period (LP, days from inoculation to the appearance of the first pustule) and lesion length (LL, cm, recorded 75 days post-inoculation) were measured for each genotype using a graduated scale. The coefficient of infection (CI) was calculated as the product of disease severity and a constant assigned to infection type (immune = 0, R = 0.2, MR = 0.4, M = 0.6, MS = 0.8, S = 1.0) [48], and the mean of the five within-season readings gave the average coefficient of infection (ACI). The area under the disease progress curve (AUDPC) was calculated from severity readings across the season following Wilcoxson et al. [49]:

*AUDPC* = Σ [(*D_i_* + *D_i_*_+1_)/2] × (*T_i_*_+1_ − *T_i_*)

where D is disease severity at a given date, and T is the time interval, in days, between two consecutive observations. Relative AUDPC (rAUDPC) was expressed as a percentage of the maximum possible AUDPC over the assessment period. Final disease severity (FDS) recorded at the last scoring date was used to classify genotypes into seven reaction classes: immune (IM), highly resistant (HR), resistant (R), moderately resistant (MR), moderately susceptible (MS), susceptible (S) and highly susceptible (HS).

### 5.5. Molecular Characterization of Adult Plant Resistance Genes

All 200. genotypes were genotyped for the presence of *Yr18*, *Yr29* and *Yr46* using the linked SSR markers *csLV34*, *wmc44* and *gwm192*, respectively. Marker sequences and chromosomal positions were retrieved from the GrainGenes database (http://wheat.pw.usda.gov/ accessed on 16 March 2025), and primers were custom-synthesized (Eurofins Genomics India Pvt. Ltd., Bengaluru, India). The gene-linked SSR marker *csLV34* is a co-dominant STS marker developed from the *Yr18/Lr34* candidate-gene sequence and is therefore very tightly (functionally) associated with the gene, whereas *wmc44* and *gwm192* are genomic SSR markers mapped in flanking linkage with *Yr29* and *Yr46*, respectively, and are not derived from the genes themselves. Genomic DNA was extracted from fresh, tender leaves of 20–25-day-old field-grown seedlings using the CTAB method [50]. Briefly, leaf tissue ground in liquid nitrogen was incubated in pre-heated (65 °C) CTAB extraction buffer containing 0.2% β-mercaptoethanol for 1 h, and DNA was purified by chloroform:isoamyl alcohol (24:1) extraction, isopropanol precipitation, and washing with 70% ethanol. Dried pellets were resuspended in TE buffer (10 mM Tris, 1 mM EDTA, pH 8.0), treated with RNase-A (Sigma-Aldrich, Karnataka, India) at 37 °C for 2–3 h to remove RNA contamination, and DNA quality was verified by 0.8% agarose gel electrophoresis and NanoDrop spectrophotometry (Analytik Jena ScanDrop2, Jena, Germany). Working DNA stocks were normalized to 20–25 ng µL^−1^. PCR amplification was carried out in a 10 µL reaction volume containing 1 µL template DNA, 5 µLGoTaq^®^ Green Master Mix, 0.5 µL each of forward and reverse primers (10 µM) (Table 3), and 3 µL nuclease-free water, using a Biometra TRIO thermal cycler (Analytik Jena, Jena, Germany). The cycling profile comprised initial denaturation at 94 °C for 5 min; 35–45 cycles of 94 °C for 30 s, primer-specific annealing temperature for 1 min, and 72 °C for 30 s; a final extension at 72 °C for 10 min; and a 4 °C hold. Amplicons were resolved on 1.5–2.5% agarose gels in 1× TBE buffer containing ethidium bromide (0.5 µg ml^−1^), visualized under UV light (VilberLourmat gel documentation system, Eberhardzell, Germany), and scored as present (1) or absent (0) relative to a 100 bp DNA ladder.

### 5.6. Statistical Analysis

Correlations between final disease severities across seasons were computed using SAS 9.2 (SAS Institute, Cary, NC, USA). Analysis of variance was used to test the effects of individual and combined APR genes on FDS, ACI and AUDPC. Relationships among AUDPC, ACI, latent period, lesion length and the number of APR genes present were examined by Pearson correlation and regression, and principal component analysis (PCA) was used to visualize genotype groupings based on disease-related traits, using the *metan* package in R software (R version 4.x). PCA was performed on the correlation matrix of five disease-related traits (latent period, lesion length, ACI, AUDPC and rAUDPC) using genotype mean values averaged over the three field seasons; variables were standardised (unit variance scaling) prior to ordination, and the first two principal components, retained on the basis of eigenvalues >1, were used to construct the biplot.

## Figures and Tables

**Table 1:** Latent period, lesion length, average coefficient of infection (ACI), AUDPC, rAUDPC, postulated *Yr* genes and disease reaction category for the 200 wheat genotypes under field conditions over three consecutive years (2021–2024).

Genotype	Latent Period (Days, Mean ± SD)	Lesion Length (cm)	ACI (%, Mean ± SD)	AUDPC	rAUDPC (%)	YrGenes	Category
CWI 272	12 ± 0.00	21.9 ± 1.39	66.7 ± 11.50	625.33 ± 217.00	52.55	*Yr29*	HS
CWI13832	13 ± 1.00	16.1 ± 6.79	60 ± 20.00	505.17 ± 292.80	42.45	*Yr29*	HS
CWI 13840	18 ± 3.61	6.9 ± 6.58	24 ± 31.20	240.8 ± 337.50	20.24	*Yr29*	MS
CWI 13841	17 ± 3.00	8 ± 5.36	28 ± 28.00	271.13 ± 314.50	22.78	*Yr18+Yr29*	MS
CWI 13496	23 ± 1.00	1.6 ± 0.23	0.2 ± 0.00	2.8 ± 0.00	0.24	*Yr29*	HR
BW16110	16.3 ± 3.79	14.7 ± 5.65	50.7 ± 25.70	406 ± 230.50	34.12	*Yr29*	S
BW16113	17 ± 4.36	9.3 ± 10.37	28.1 ± 45.00	438.67 ± 350.10	36.86	*Yr18+Yr29*	MS
BW 17360	19.3 ± 5.51	4.9 ± 2.82	22 ± 17.30	161.7 ± 138.70	13.59	*Yr18+Yr29*	MS
BW 17364	19 ± 5.20	8.1 ± 8.83	28 ± 28.00	261.33 ± 173.50	21.96	*Yr29*	MS
BW 17365	15.7 ± 4.62	10.7 ± 7.57	42.7 ± 30.00	417.67 ± 309.1	35.10	*Yr46*	S
BW 17369	15.7 ± 6.35	12.3 ± 7.26	42.7 ± 30.00	378.23 ± 302.50	31.78	*Yr29+Yr46*	S
BW 17370	23 ± 2.00	1.4 ± 0.23	0.2 ± 0.00	2.8 ± 0.00	0.24	*Yr18+Yr29*	HR
BW 17372	15.3 ± 2.31	12 ± 10.85	73.3 ± 11.50	714 ± 178.70	60.00	*Yr29+Yr46*	HS
BW 17373	24 ± 1.00	1.2 ± 0.17	0.2 ± 0.00	2.8 ± 0.00	0.24	*Yr29*	HR
BW 17376	23.3 ± 1.53	1.6 ± 0.23	0.2 ± 0.00	2.8 ± 0.00	0.24	*Yr29*	HR
BW 17378	18 ± 3.00	9.6 ± 6.18	18.7 ± 12.20	156.33 ± 84.70	13.14	*Yr18+Yr29+Yr46*	MR
BW 17379	14 ± 3.46	16.7 ± 3.06	66.7 ± 23.10	597.33 ± 301.70	50.20	*Yr18+Yr29*	HS
BW 17381	15.7 ± 1.53	13.9 ± 5.95	50.7 ± 25.70	434 ± 355.00	36.47	*Yr18+Yr29*	S
BW 17382	23.7 ± 2.31	1.1 ± 0.12	0.1 ± 0.10	1.87 ± 1.60	0.16	*Yr18+Yr29*	HR
BW 17383	17 ± 5.29	5.8 ± 5.37	36 ± 26.20	301 ± 249.20	25.29	*Yr18+Yr29*	MS
BW 17387	16 ± 3.46	16.2 ± 0.81	36 ± 22.30	294 ± 105.70	24.71	*Yr18+Yr29+Yr46*	MS
BW 17393	16.3 ± 4.04	16.8 ± 4.79	53.3 ± 30.60	485.33 ± 193.10	40.78	*Yr18+Yr29+Yr46*	S
BW 17395	17.7 ± 0.58	2.1 ± 1.39	4 ± 4.00	28.7 ± 28.40	2.41	*Yr18+Yr29+Yr46*	R
BW 17396	15.3 ± 1.15	6.3 ± 4.62	22.7 ± 16.20	201.13 ± 153.50	16.90	*Yr29+Yr46*	MS
BW 17397	18.7 ± 4.04	5.1 ± 0.75	16 ± 13.90	102.67 ± 68.70	8.63	*Yr18+Yr29+Yr46*	MR
BW 17398	19.3 ± 3.79	2.4 ± 0.75	8 ± 0.00	74.67 ± 20.20	6.27	*Yr18+Yr29*	R
BW 17418	16 ± 4.36	6.7 ± 4.25	16 ± 6.90	182 ± 103.10	15.29	*Yr18+Yr29*	MR
BW 17428	16.3 ± 5.13	6.3 ± 3.50	14.7 ± 6.10	119 ± 49.00	10.00	*Yr18+Yr29*	MR
BW 17386	17.3 ± 2.52	8.3 ± 5.92	22.7 ± 8.30	198.33 ± 77.40	16.67	*Yr18+Yr29*	MS
BW 17388	14 ± 2.00	12.2 ± 6.56	37.3 ± 20.50	310.33 ± 84.70	26.08	*Yr18+Yr29*	MS
BW 17361	14 ± 1.00	13.4 ± 6.18	44 ± 14.40	303.33 ± 29.10	25.49	*Yr29*	S
BW 17440	15 ± 2.65	18.4 ± 3.19	60 ± 20.00	555.33 ± 194.20	46.67	*Yr29*	HS
BW37110	13 ± 1.00	20.1 ± 2.85	80 ± 0.00	718.67 ± 221.50	60.39	*Yr46*	HS
BW21934	14.3 ± 1.53	19.3 ± 4.05	60 ± 0.00	583.33 ± 40.40	49.02	*Yr29+Yr46*	HS
CWI 13091	14 ± 0.00	11.6 ± 5.80	44 ± 14.40	445.67 ± 282.60	37.45	*Yr29+Yr46*	S
CW 13101	12.3 ± 0.58	15.1 ± 7.60	40 ± 20.00	345.33 ± 198.60	29.02	*Yr29*	S
CWI 13106	15.3 ± 1.53	9.2 ± 5.19	22.7 ± 16.20	175.47 ± 130.10	14.75	*Yr29+Yr46*	MS
CWI 13118	14 ± 2.00	8.7 ± 2.65	37.3 ± 4.60	291.67 ± 34.50	24.51	*Yr46*	MS
BW 18870	14.7 ± 0.58	12.6 ± 7.34	41.3 ± 16.20	429.33 ± 295.80	36.08	*Yr46*	S
CWI 38039	14.3 ± 2.52	14.9 ± 6.98	44 ± 14.40	445.67 ± 282.6	37.45	0	S
CWI 38038	14.3 ± 2.08	15.6 ± 8.14	50.7 ± 25.70	455 ± 190.50	38.24	*Yr46*	S
CWI 38037	15 ± 1.00	12.3 ± 8.08	44 ± 31.70	413 ± 278.90	34.71	*Yr46*	S
CWI 38023	16.3 ± 2.89	12.8 ± 9.53	45.3 ± 32.30	359.33 ± 253.60	30.20	*Yr46*	S
CWI 38022	15.3 ± 1.53	5.9 ± 1.59	26.7 ± 16.70	238 ± 124.40	20.00	*Yr46*	MS
CWI 9739	18.3 ± 6.51	4 ± 2.86	12.1 ± 10.50	89.6 ± 86.10	7.53	*Yr18+Yr46*	MR
BW 31659	16.3 ± 5.77	9.9 ± 6.37	38.7 ± 22.00	273 ± 133.40	22.94	*Yr18+Yr29+Yr46*	MS
BW 1899	14.7 ± 3.06	10.6 ± 4.61	44 ± 14.40	317.33 ± 56.60	26.67	*Yr18+Yr29*	S
BW 2328	17 ± 4.58	9.6 ± 8.86	37.3 ± 37.00	364.47 ± 472.50	30.63	*Yr46*	MS
BW 6213	15 ± 2.65	13.2 ± 0.81	46.7 ± 11.50	357 ± 117.80	30.00	*Yr29+Yr46*	S
CWI 38019	17.3 ± 2.08	7.5 ± 4.79	36 ± 26.20	341.13 ± 267.20	28.67	*Yr29+Yr46*	MS
BW 8323	17.7 ± 4.73	9.3 ± 6.40	25.3 ± 12.90	196 ± 103.60	16.47	*Yr18*	MS
BW 8329	18 ± 5.57	5.3 ± 1.65	12 ± 6.90	87.97 ± 61.00	7.39	*Yr18*	MR
BW 8330	20 ± 4.58	6.4 ± 5.08	12 ± 6.90	91.47 ± 57.20	7.69	*Yr29*	MR
BW 8331	23.3 ± 1.53	3.7 ± 1.73	16 ± 13.90	124.83 ± 110.10	10.49	*Yr29*	MR
BW 8332	14.3 ± 0.58	14.9 ± 3.36	40 ± 0.00	333.67 ± 17.60	28.04	*Yr18+Yr29*	S
BW 8333	21.7 ± 3.06	1.8 ± 0.4	4.1 ± 3.90	33.6 ± 30.10	2.82	*Yr18+Yr29*	R
BW 9189	12.7 ± 1.15	19.7 ± 0.85	66.7 ± 11.50	653.33 ± 106.90	54.90	*Yr29*	HS
BW 16556	21 ± 1.00	2 ± 0.00	0.7 ± 1.20	3.73 ± 6.50	0.31	*Yr18*	HR
BW 16609	13.3 ± 1.15	18 ± 3.46	66.7 ± 23.10	525 ± 181.90	44.12	0	HS
BW 15845	12.7 ± 1.15	19 ± 6.07	80 ± 0.00	770 ± 121.20	64.71	0	HS
BW 16611	13 ± 0.00	12 ± 9.54	44 ± 31.70	333.67 ± 256.80	28.04	*Yr29*	S
BW 16612	17.7 ± 0.58	5.1 ± 0.72	5.3 ± 9.20	42 ± 72.70	3.53	*Yr18+Yr29*	R
BW 16614	15.3 ± 1.53	11.8 ± 8.00	38.7 ± 22.00	331.33 ± 262.00	27.84	*Yr18*	MS
BW 16635	16.3 ± 1.53	12.2 ± 7.41	25.3 ± 12.90	198.33 ± 102.00	16.67	*Yr29*	MS
BW 15782	14.3 ± 2.52	14.7 ± 3.94	53.3 ± 11.50	497 ± 163.10	41.76	*Yr29*	S
BW 15907	17.7 ± 3.06	16.2 ± 5.12	58.7 ± 37.00	515.67 ± 384.40	43.33	*Yr29*	S
BW 15908	14.3 ± 3.21	16.1 ± 10.74	64 ± 27.70	644 ± 339.50	54.12	*Yr29*	HS
BW 16096	12.7 ± 1.15	22.3 ± 0.91	80 ± 0.00	770 ± 242.50	64.71	*Yr29*	HS
BW 16098	12.7 ± 0.58	18.7 ± 1.35	73.3 ± 11.50	688.33 ± 336.30	57.84	*Yr29*	HS
BW 16099	14.3 ± 2.52	14.7 ± 9.74	45.3 ± 25.40	403.67 ± 270.80	33.92	*Yr29*	S
BW 16101	12.7 ± 1.15	11.6 ± 8.79	46.7 ± 11.50	455 ± 273.40	38.24	*Yr29*	S
BW 16102	13.3 ± 1.53	17.6 ± 3.16	60 ± 20.00	567 ± 263.10	47.65	0	HS
BW 16103	13 ± 1.00	17.7 ± 0.58	80 ± 0.00	683.67 ± 135.80	57.45	*Yr29*	HS
BW 16104	16 ± 4.36	14 ± 9.29	38.7 ± 22.00	269.5 ± 154.80	22.65	*Yr29*	MS
BW 16106	17.3 ± 2.89	14.4 ± 11.09	21.3 ± 16.70	155.63 ± 129.60	13.08	*Yr29*	MS
BW 16107	14 ± 3.46	14.7 ± 3.06	46.7 ± 11.50	415.33 ± 125.50	34.90	*Yr18*	S
BW 16108	17 ± 5.20	12.8 ± 10.09	18.7 ± 18.50	205.8 ± 129.90	17.29	*Yr18*	MR
BW 16109	15.3 ± 3.21	21 ± 0.58	73.3 ± 11.50	630 ± 0.00	52.94	*Yr29*	HS
CWI 10963	12 ± 0.00	19.3 ± 1.65	60 ± 20.00	513.33 ± 145.70	43.14	0	HS
BW 16112	15.7 ± 2.08	15.2 ± 11.53	36 ± 38.60	281.63 ± 305.80	23.67	*Yr18*	MS
BW 1896	15 ± 2.65	17.2 ± 3.50	66.7 ± 11.50	597.33 ± 153.60	50.20	*Yr29*	HS
BW2325	15.3 ± 4.16	14.6 ± 10.88	56 ± 41.60	525 ± 408.80	44.12	*Yr29*	S
BW 2327	14.7 ± 0.58	18.3 ± 5.98	53.3 ± 23.10	550.67 ± 260.20	46.27	*Yr29*	S
BW 2663	13.7 ± 1.53	13.6 ± 4.37	60 ± 20.00	595 ± 264.20	50.00	*Yr29*	HS
BW 6251	14.3 ± 1.53	13.9 ± 3.50	46.7 ± 11.50	415.33 ± 125.50	34.90	*Yr18*	S
BW 8319	15 ± 2.65	10.8 ± 8.84	22.7 ± 8.30	219.33 ± 155.30	18.43	*Yr18+Yr46*	MS
BW 8320	15.3 ± 3.06	15.1 ± 4.33	46.7 ± 23.10	385 ± 245.00	32.35	*Yr46*	S
BW 8321	15 ± 2.65	9.6 ± 2.89	33.3 ± 11.50	298.67 ± 47.60	25.10	*Yr29+Yr46*	MS
BW 8322	18 ± 2.00	3.8 ± 3.06	16 ± 13.90	168.93 ± 193.20	14.20	*Yr18+Yr29+Yr46*	MR
BW 8325	19 ± 4.00	11.8 ± 7.25	16 ± 0.00	150.5 ± 41.30	12.65	*Yr18+Yr29*	MR
BW 8334	15 ± 3.61	15.8 ± 2.01	53.3 ± 11.50	555.33 ± 217.00	46.67	*Yr18*	S
BW 8335	14 ± 1.00	19.3 ± 1.53	66.7 ± 11.50	571.67 ± 248.30	48.04	*Yr29*	HS
BW 8336	14.7 ± 2.08	15.8 ± 10.48	36 ± 26.20	312.67 ± 248.50	26.27	*Yr29*	MS
BW 8337	13.7 ± 2.08	17.1 ± 2.71	53.3 ± 30.60	452.67 ± 249.00	38.04	*Yr18+Yr29*	S
BW 8341	12.3 ± 0.58	20.3 ± 2.65	60 ± 0.00	597.33 ± 157.40	50.20	*Yr29*	HS
BW 8950	15 ± 3.61	16.7 ± 3.27	46.7 ± 11.50	485.33 ± 251.40	40.78	*Yr29*	S
BW 9242	15.7 ± 4.73	19.8 ± 2.76	60 ± 0.00	487.67 ± 91.40	40.98	*Yr29+Yr46*	HS
BW 12202	16.7 ± 4.73	15.7 ± 9.50	49.3 ± 37.20	476 ± 371.20	40.00	*Yr29+Yr46*	S
BW 29910	17 ± 6.08	15 ± 7.24	49.3 ± 37.20	557.67 ± 493.50	46.86	*Yr29+Yr46*	S
T 3862	17.3 ± 6.81	13.4 ± 8.79	49.3 ± 37.20	541.33 ± 441.00	45.49	*Yr29+Yr46*	S
CWI 36473	15.7 ± 3.21	3.1 ± 0.51	13.3 ± 4.60	109.9 ± 78.40	9.24	*Yr46*	MR
CWI 41172	16.7 ± 4.04	9.4 ± 11.76	32 ± 41.60	322.7 ± 448.50	27.12	*Yr46*	MS
CWI 32610	13 ± 1.00	19.2 ± 2.93	66.7 ± 11.50	665 ± 344.70	55.88	*Yr29*	HS
CWI 85928	12.7 ± 1.15	16.9 ± 2.82	40 ± 0.00	420 ± 121.20	35.29	*Yr29+Yr46*	S
BW 35109	0 ± 0.00	0 ± 0.00	0 ± 0.00	0 ± 0.00	0.00	*Yr18+Yr29+Yr46*	I
BW 35110	0 ± 0.00	0 ± 0.00	0 ± 0.00	0 ± 0.00	0.00	*Yr18+Yr29+Yr46*	I
BW 35112	0 ± 0.00	0 ± 0.00	0 ± 0.00	0 ± 0.00	0.00	*Yr18+Yr29+Yr46*	I
BW 35114	0 ± 0.00	0 ± 0.00	0 ± 0.00	0 ± 0.00	0.00	*Yr29+Yr46*	I
BW 35116	0 ± 0.00	0 ± 0.00	0 ± 0.00	0 ± 0.00	0.00	*Yr29+Yr46*	I
DW 7958	13.3 ± 1.15	15.3 ± 3.21	46.7 ± 11.50	443.33 ± 145.70	37.25	*Yr46*	S
DW 7963	15.3 ± 3.06	15.2 ± 7.88	37.3 ± 4.60	317.33 ± 168.20	26.67	*Yr46*	MS
BW 27274	14 ± 2.65	14 ± 9.36	50.7 ± 25.70	445.67 ± 282.60	37.45	*Yr29+Yr46*	S
BW 33914	14.7 ± 1.15	17 ± 4.12	46.7 ± 30.60	609 ± 388.90	51.18	*Yr46*	S
BW 32061	17 ± 4.58	14.1 ± 6.30	40 ± 20.00	350 ± 124.40	29.41	*Yr18+Yr29+Yr46*	S
BW 37109	15.7 ± 5.51	17.3 ± 5.13	60 ± 20.00	616 ± 379.00	51.76	*Yr29+Yr46*	HS
CWI 51010	14 ± 2.00	14.7 ± 7.21	44 ± 31.70	389.67 ± 208.90	32.75	*Yr46*	S
CWI 13085	14.3 ± 1.53	13.8 ± 7.80	40 ± 34.60	415.33 ± 367.80	34.90	*Yr29+Yr46*	S
BW 44529	15 ± 0.00	16.3 ± 0.85	53.3 ± 23.10	555.33 ± 428.40	46.67	*Yr29+Yr46*	S
BW 44334	16 ± 3.46	10.6 ± 6.70	44 ± 14.40	462 ± 268.20	38.82	*Yr46*	S
BW 44335	16.3 ± 3.06	7.2 ± 4.50	45.3 ± 32.30	429.33 ± 372.90	36.08	ND	S
BW 44336	14.3 ± 1.53	18.3 ± 3.35	60 ± 20.00	555.33 ± 280.10	46.67	*Yr46*	HS
BW 44337	14.7 ± 4.62	19.4 ± 3.01	53.3 ± 23.10	513.33 ± 291.40	43.14	*Yr46*	S
BW 44338	13.7 ± 0.58	16.1 ± 5.60	40 ± 34.60	396.67 ± 385.50	33.33	*Yr46*	S
CWI 29059	15.3 ± 0.58	10.6 ± 7.34	28 ± 6.90	219.33 ± 71.80	18.43	*Yr46*	MS
CWI 7130	13.3 ± 2.31	14.2 ± 3.29	46.7 ± 11.50	401.33 ± 138.10	33.73	*Yr29+Yr46*	S
CWI 8045	16 ± 2.65	7.1 ± 5.95	29.3 ± 18.50	242.67 ± 127.00	20.39	*Yr46*	MS
CWI 9737	18.3 ± 5.51	9 ± 8.76	28 ± 28.00	264.13 ± 268.40	22.20	*Yr29+Yr46*	MS
CWI 91687	15 ± 3.61	13.7 ± 9.74	66.7 ± 23.10	625.33 ± 287.00	52.55	*Yr29+Yr46*	HS
CWI 98580	14.3 ± 1.53	13.4 ± 6.26	53.3 ± 11.50	529.67 ± 228.60	44.51	*Yr18+Yr46*	S
CWI 98601	16 ± 1.00	12.9 ± 6.24	25.3 ± 12.90	179.67 ± 87.50	15.10	*Yr18+Yr29+Yr46*	MS
CWI 98609	16 ± 3.00	11.7 ± 5.38	44 ± 14.40	392 ± 328.00	32.94	*Yr18+Yr29+Yr46*	S
CWI 98629	15.7 ± 1.53	10.1 ± 8.68	50.7 ± 25.70	443.33 ± 343.60	37.25	*Yr29+Yr46*	S
CWI 98662	16.3 ± 4.51	12.4 ± 7.89	22.7 ± 16.20	240.33 ± 222.50	20.20	YR29+YR46	MS
CWI 75596	15.3 ± 5.77	17.6 ± 6.01	49.3 ± 37.20	536.67 ± 409.90	45.10	*Yr18+Yr46*	S
CWI 98753	14 ± 1.00	14.1 ± 8.75	53.3 ± 30.60	449.17 ± 351.90	37.75	*Yr29+Yr46*	S
CWI 98815	15.3 ± 1.53	10.3 ± 8.5	50.7 ± 25.70	464.33 ± 392.90	39.02	*Yr18+Yr46*	S
CWI 98817	15.7 ± 2.52	13.3 ± 8.82	36 ± 26.20	345.8 ± 331.40	29.06	*Yr29+Yr46*	MS
CWI 98886	14 ± 1.73	19.4 ± 2.89	40 ± 20.00	378 ± 285.90	31.76	*Yr18+Yr29+Yr46*	S
CWI 98913	16 ± 2.00	16 ± 2.65	21.3 ± 18.00	242.67 ± 103.20	20.39	*Yr18+Yr29+Yr46*	MS
CWI 98928	13 ± 1.00	14.6 ± 4.37	46.7 ± 23.10	494.67 ± 313.20	41.57	*Yr46*	S
BW 8947	13.7 ± 0.58	19 ± 3.16	53.3 ± 11.50	476 ± 91.80	40.00	*Yr18+Yr29+Yr46*	S
CWI 99449	13.7 ± 1.53	12.3 ± 9.79	26.7 ± 30.50	276.27 ± 321.20	23.22	*Yr29+Yr46*	MS
CWI 99459	14 ± 2.65	21.4 ± 1.40	66.7 ± 23.10	616 ± 301.20	51.76	*Yr46*	HS
BW 15906	16.7 ± 4.04	12.1 ± 8.59	36 ± 26.20	326.67 ± 204.00	27.45	*Yr46*	MS
CWI 99812	15.7 ± 2.08	12.4 ± 3.67	46.7 ± 11.50	406 ± 77.90	34.12	*Yr18+Yr46*	S
CWI 41166	20.3 ± 3.06	5.7 ± 4.67	12 ± 6.90	107.33 ± 76.80	9.02	*Yr18+Yr46*	MR
T 1074	20 ± 3.46	6.2 ± 6.58	18.7 ± 18.50	130.67 ± 117.20	10.98	*Yr46*	MR
CWI 41396	17.7 ± 3.51	8.8 ± 11.72	34.7 ± 39.50	284.67 ± 299.40	23.92	*Yr46*	MS
T 1337	19 ± 6.56	9.8 ± 11.72	32 ± 41.60	250.13 ± 329.00	21.02	*Yr46*	MS
CWI 41399	21.7 ± 1.15	4 ± 0.89	14.7 ± 6.10	179.67 ± 150.80	15.10	*Yr29+Yr46*	MR
T 1659	17.3 ± 4.51	11.2 ± 5.89	36 ± 26.20	280 ± 188.00	23.53	*Yr29+Yr46*	MS
DW 895	15 ± 3.61	15.9 ± 4.18	42.7 ± 36.10	387.8 ± 409.10	32.59	*Yr46*	S
19th HTWYT2	22.7 ± 2.89	2.8 ± 1.08	6.7 ± 2.30	46.2 ± 24.40	3.88	*Yr29+Yr46*	R
19th HTWYT3	22 ± 3.00	2.1 ± 1.06	9.3 ± 6.10	66.27 ± 52.20	5.57	*Yr29+Yr46*	R
19th HTWYT4	19.7 ± 3.21	4.2 ± 2.54	21.3 ± 9.20	163.33 ± 102.00	13.73	*Yr46*	MS
19th HTWYT5	14.3 ± 4.04	15.6 ± 1.40	42.7 ± 4.60	345.33 ± 130.10	29.02	ND	S
19^th^HTWYT6	19 ± 2.65	4.3 ± 0.58	18.7 ± 12.20	144.67 ± 118.00	12.16	*Yr46*	MR
19th HTWYT7	20 ± 3.61	2 ± 0.30	16 ± 13.90	135.33 ± 125.30	11.37	*Yr29+Yr46*	MR
19th HTWYT8	19 ± 1.73	2.6 ± 2.40	4.7 ± 3.10	46.2 ± 45.80	3.88	*Yr18+Yr29+Yr46*	R
19th HTWYT9	18 ± 5.20	6.8 ± 7.17	12 ± 6.90	121.8 ± 106.70	10.24	*Yr18+Yr29+Yr46*	MR
19th HTWYT10	16.3 ± 4.93	10.3 ± 7.21	32 ± 24.30	263.67 ± 78.70	22.16	*Yr29+Yr46*	MS
41st ESWYT 111	17.7 ± 5.51	10 ± 9.36	34.7 ± 39.50	343 ± 431.60	28.82	*Yr46*	MS
41st ESWYT 112	23 ± 1.00	3.5 ± 3.06	2.9 ± 4.40	23.57 ± 34.20	1.98	*Yr18+Yr46*	HR
41st ESWYT 113	22.7 ± 1.53	2.9 ± 1.40	4.1 ± 3.90	33.6 ± 27.60	2.82	*Yr46*	R
41st ESWYT 114	22 ± 3.00	2.9 ± 1.91	9.3 ± 6.10	70.7 ± 60.40	5.94	*Yr29+Yr46*	R
41st ESWYT 115	18.3 ± 3.06	2.2 ± 1.56	10.7 ± 4.60	82.13 ± 38.10	6.90	*Yr29+Yr46*	MR
41st ESWYT 116	19.3 ± 4.73	8.4 ± 9.44	25.3 ± 30.00	216.3 ± 297.90	18.18	*Yr46*	MS
41st ESWYT 117	17 ± 4.58	11.7 ± 9.58	32 ± 41.60	331.8 ± 440.60	27.88	*Yr18+Yr29+Yr46*	MS
41st ESWYT 118	16.3 ± 4.93	9 ± 6.77	22.7 ± 16.20	178.5 ± 127.30	15.00	*Yr18+Yr29+Yr46*	MS
41st ESWYT 119	20 ± 5.57	7.7 ± 10.43	28.1 ± 45.00	286.77 ± 479.20	24.10	*Yr29+Yr46*	MS
41st ESWYT 120	18 ± 3.61	9.8 ± 12.07	34.7 ± 39.50	413 ± 552.60	34.71	*Yr18+Yr29+Yr46*	MS
51st ESWYT 121	16.7 ± 5.69	9.2 ± 10.20	34.7 ± 39.50	399.47 ± 563.60	33.57	*Yr18+Yr29+Yr46*	MS
51st ESWYT 122	17.7 ± 4.16	8.4 ± 5.75	21.3 ± 16.70	170.33 ± 93.00	14.31	*Yr29+Yr46*	MS
51st ESWYT 123	14.7 ± 3.06	9.9 ± 10.48	42.7 ± 36.10	476 ± 512.80	40.00	*Yr29+Yr46*	S
51st ESWYT 124	16.7 ± 5.51	13.3 ± 9.72	42.7 ± 36.10	469.47 ± 520.80	39.45	*Yr18+Yr29*	S
51st ESWYT 125	18 ± 3.61	3.3 ± 3.45	30 ± 24.60	264.83 ± 247.10	22.25	*Yr29+Yr46*	MS
51st ESWYT 126	19.3 ± 3.06	10.9 ± 8.06	28 ± 20.80	219.33 ± 154.40	18.43	*Yr29+Yr46*	MS
51st ESWYT 127	18.3 ± 4.51	11.7 ± 9.17	18.7 ± 18.50	170.33 ± 156.60	14.31	*Yr29+Yr46*	MR
51st ESWYT 128	15.3 ± 1.53	21.9 ± 0.85	66.7 ± 23.10	625.33 ± 287.00	52.55	*Yr18+Yr29+Yr46*	HS
51st ESWYT 129	15 ± 1.00	16.9 ± 2.08	53.3 ± 11.50	529.67 ± 228.60	44.51	*Yr18+Yr46*	S
51st ESWYT 130	18.7 ± 2.52	13.7 ± 7.22	22.7 ± 16.20	184.33 ± 139.00	15.49	ND	MS
51st ESWYT 131	20 ± 4.36	8 ± 8.54	22.7 ± 16.20	177.8 ± 143.10	14.94	*Yr29*	MS
51st ESWYT 132	14.7 ± 3.06	11.9 ± 11.11	40 ± 20.00	340.67 ± 205.00	28.63	*Yr29*	S
51st ESWYT 133	16 ± 2.00	15.8 ± 3.64	33.3 ± 11.50	301 ± 178.60	25.29	*Yr46*	MS
51st ESWYT 134	20.3 ± 3.06	2 ± 0.89	6 ± 3.5.00	50.87 ± 43.90	4.27	*Yr29+Yr46*	R
51st ESWYT 135	23.3 ± 1.15	1.5 ± 0.40	0.2 ± 0.00	2.8 ± 0.00	0.24	*Yr29+Yr46*	HR
51st ESWYT 136	21.7 ± 6.51	7 ± 5.76	8 ± 0.00	72.57 ± 22.30	6.10	*Yr18+Yr29+Yr46*	R
51st ESWYT 137	16 ± 2.65	13.6 ± 4.21	26.7 ± 11.50	280 ± 183.10	23.53	*Yr29*	MS
51st ESWYT 138	15.3 ± 3.06	11.4 ± 6.83	23.3 ± 15.30	261.33 ± 124.50	21.96	*Yr46*	MS
51st ESWYT 139	19 ± 4.36	5.7 ± 4.93	9.3 ± 9.20	91 ± 91.50	7.65	*Yr46*	R
51st ESWYT 140	21 ± 3.61	2.9 ± 1.40	0.1 ± 0.10	494.67 ± 313.20	41.57	ND	HR
51st ESWYT 141	22 ± 3.61	5.6 ± 5.61	4.1 ± 3.90	43.4 ± 49.10	3.65	*Yr18+Yr29+Yr46*	R
51st ESWYT 142	22.3 ± 4.16	6 ± 4.60	6.7 ± 2.30	45.03 ± 17.20	3.78	*Yr29+Yr46*	R
51st ESWYT 143	20.7 ± 2.31	2.7 ± 0.91	10.7 ± 8.30	102.67 ± 81.10	8.63	*Yr29+Yr46*	MR
51st ESWYT 144	18.7 ± 5.86	8.1 ± 6.40	17.3 ± 19.70	196 ± 255.50	16.47	*Yr18+Yr29+Yr46*	MR
51st ESWYT 145	0 ± 0.00	0 ± 0.00	0 ± 0.00	0 ± 0.00	0.00	*Yr29+Yr46*	I
51st ESWYT 146	0 ± 0.00	0 ± 0.00	0 ± 0.00	0 ± 0.00	0.00	*Yr29+Yr46*	I
51st ESWYT 147	0 ± 0.00	0 ± 0.00	0 ± 0.00	0 ± 0.00	0.00	*Yr29+Yr46*	I
51st ESWYT 148	17.7 ± 3.06	4.7 ± 2.03	29.3 ± 20.10	266 ± 175.40	22.35	*Yr29+Yr46*	MS
51st ESWYT 149	17.7 ± 1.53	5 ± 1.30	28 ± 17.40	261.33 ± 167.00	21.96	*Yr29+Yr46*	MS
Mean	15.9 ± 0.85	10.67 ± 2.29	66.7 ± 11.50	1190 ± 121.20	100.00		
SD	5.55	7.07					
SE	0.39	0.49					
CV	38.72	80.45					
CD	0.39	0.80					

ACI, average coefficient of infection; AUDPC, area under the disease progress curve; rAUDPC, relative AUDPC. Category: I, immune; HR, highly resistant; R, resistant; MR, moderately resistant; MS, moderately susceptible; S, susceptible; HS, highly susceptible; Susceptible checks (Agra Local, Kharchia 65, PBW 343, A-9-30-1) recorded 90% final disease severity in all three seasons; ND, not detected; SD: standard deviation; SE: standard error; CV: coefficient of variation; CD: critical difference (5%). Wheat genotypes i.e., 51st ESWYT 121 to 51st ESWYT 121 were originally derived from the 41st ESWYT nursery.

**Table 2:** Seedling infection type against five stripe rust pathotypes, adult-plant ACI and AUDPC, and molecular postulation of adult plant resistance gene(s) for the 200 wheat genotypes.

Genotype	46S119	110S119	238S119	110S84	47S103	ACI (%)	AUDPC	MAS Result (*Yr*Genes)
CWI 272	3+	3+	3+	3+	3+	66.7 ± 11.50	625.33 ± 217.00	*Yr29*
CWI13832	3−	3−	3−	3−	3+	60 ± 20.00	505.17 ± 292.80	*Yr29*
CWI 13840	3+	3+	3+	3+	3+	24 ± 31.20	240.8 ± 337.50	*Yr29*
CWI 13841	3	3	3+	3+	3+	28 ± 28.00	271.13 ± 314.50	*Yr18*+*Yr29*
CWI 13496	0;	0;	0;	1N	0,	0.2 ± 0.00	2.8 ± 0.00	*Yr29*
BW16110	0,	0,	3	3+	0,	50.7 ± 25.70	406 ± 230.50	*Yr29*
BW16113	3+	3+	3+	3+	3+	28.1 ± 45.00	438.67 ± 350.10	*Yr18*+*Yr29*
BW 17360	3	3	3+	3+	3+	22 ± 17.30	161.7 ± 138.70	*Yr18*+*Yr29*
BW 17364	3	3	3+	3+	3+	28 ± 28.00	261.33 ± 173.50	*Yr29*
BW 17365	3+	3+	3+	3+	3+	42.7 ± 30.00	417.67 ± 309.1	*Yr46*
BW 17369	3N	3N	3+	3+	3+	42.7 ± 30.00	378.23 ± 302.50	*Yr29*+*Yr46*
BW 17370	0;	0;	3+	0;	0;	0.2 ± 0.00	2.8 ± 0.00	*Yr18*+*Yr29*
BW 17372	3+	3+	3+	3+	3+	73.3 ± 11.50	714 ± 178.70	*Yr29*+*Yr46*
BW 17373	3−	3−	0;	0;	3+	0.2 ± 0.00	2.8 ± 0.00	*Yr29*
BW 17376	0;	0;	3+	0;	3+	0.2 ± 0.00	2.8 ± 0.00	*Yr29*
BW 17378	3	3	3+	0;	3+	18.7 ± 12.20	156.33 ± 84.70	*Yr18*+*Yr29*+*Yr46*
BW 17379	;N	;N	3+	3+	3+	66.7 ± 23.10	597.33 ± 301.70	*Yr18*+*Yr29*
BW 17381	3+	3+	3+	3+	3+	50.7 ± 25.70	434 ± 355.00	*Yr18*+*Yr29*
BW 17382	0;	0;	0;	;−	0;	0.1 ± 0.10	1.87 ± 1.60	*Yr18*+*Yr29*
BW 17383	3+	3+	3+	0;	3+	36 ± 26.20	301 ± 249.20	*Yr18*+*Yr29*
BW 17387	3−	3−	3+	3+	0;	36 ± 22.30	294 ± 105.70	*Yr18*+*Yr29*+*Yr46*
BW 17393	3−	3−	3+	3+	3+	53.3 ± 30.60	485.33 ± 193.10	*Yr18*+*Yr29*+*Yr46*
BW 17395	0;	0;	3+	3+	0;	4 ± 4.00	28.7 ± 28.40	*Yr18*+*Yr29*+*Yr46*
BW 17396	1N	1N	3+	3+	0;	22.7 ± 16.20	201.13 ± 153.50	*Yr29*+*Yr46*
BW 17397	3	3	3−	3−	3+	16 ± 13.90	102.67 ± 68.70	*Yr18*+*Yr29*+*Yr46*
BW 17398	3+	3+	3+	3+	3+	8 ± 0.00	74.67 ± 20.20	*Yr18*+*Yr29*
BW 17418	3+	3+	3+	3+	3+	16 ± 6.90	182 ± 103.10	*Yr18*+*Yr29*
BW 17428	;	;	0;	0;	3+	14.7 ± 6.10	119 ± 49.00	*Yr18*+*Yr29*
BW 17386	3+	3+	0;	3+	3+	22.7 ± 8.30	198.33 ± 77.40	*Yr18*+*Yr29*
BW 17388	0;	0;	3−	3+	3−	37.3 ± 20.50	310.33 ± 84.70	*Yr18*+*Yr29*
BW 17361	3+	3+	3−	3+	3+	44 ± 14.40	303.33 ± 29.10	*Yr29*
BW 17440	3+	3+	3+	3+	3+	60 ± 20.00	555.33 ± 194.20	*Yr29*
BW37110	0;	0;	3+	3+	3+	80 ± 0.00	718.67 ± 221.50	*Yr46*
BW21934	3+	3+	3+	3+	3+	60 ± 0.00	583.33 ± 40.40	*Yr29*+*Yr46*
CWI 13091	3+	3+	3+	3+	3+	44 ± 14.40	445.67 ± 282.60	*Yr29*+*Yr46*
CW 13101	3+	3+	3	3+	3+	40 ± 20.00	345.33 ± 198.60	*Yr29*
CWI 13106	3+	3+	3+	3+	3+	22.7 ± 16.20	175.47 ± 130.10	*Yr29*+*Yr46*
CWI 13118	3+	3+	3+	0;	0;	37.3 ± 4.60	291.67 ± 34.50	*Yr46*
BW 18870	3+	3+	3+	3+	3+	41.3 ± 16.20	429.33 ± 295.80	*Yr46*
CWI 38039	3+	3+	3	3	3+	44 ± 14.40	445.67 ± 282.6	0
CWI 38038	3+	3+	3	3−	3	50.7 ± 25.70	455 ± 190.50	*Yr46*
CWI 38037	3+	3+	3	3−	3+	44 ± 31.70	413 ± 278.90	*Yr46*
CWI 38023	2−N	2−N	3−	3−	3−	45.3 ± 32.30	359.33 ± 253.60	*Yr46*
CWI 38022	2−N	2−N	2−	3	3−	26.7 ± 16.70	238 ± 124.40	*Yr46*
CWI 9739	3+	3+	3+	3+	3+	12.1 ± 10.50	89.6 ± 86.10	*Yr18*+*Yr46*
BW 31659	3+	3+	3+	3+	0;	38.7 ± 22.00	273 ± 133.40	*Yr18*+*Yr29*+*Yr46*
BW 1899	3+	3+	3+	3+	3+	44 ± 14.40	317.33 ± 56.60	*Yr18*+*Yr29*
BW 2328	;N	;N	;N	3	2−	37.3 ± 37.00	364.47 ± 472.50	*Yr46*
BW 6213	3+	3+	3+	3+	3+	46.7 ± 11.50	357 ± 117.80	*Yr29*+*Yr46*
CWI 38019	0;	0;	3+	3+	3+	36 ± 26.20	341.13 ± 267.20	*Yr29*+*Yr46*
BW 8323	0;	0;	3+	3+	0;	25.3 ± 12.90	196 ± 103.60	*Yr18*
BW 8329	3+	3+	3+	3+	0;	12 ± 6.90	87.97 ± 61.00	*Yr18*
BW 8330	0;	0;	3+	3+	0;	12 ± 6.90	91.47 ± 57.20	*Yr29*
BW 8331	3+	3+	3+	3+	0;	16 ± 13.90	124.83 ± 110.10	*Yr29*
BW 8332	3−	3−	3+	3+	3+	40 ± 0.00	333.67 ± 17.60	*Yr18*+*Yr29*
BW 8333	3+	3+	3+	3	3+	4.1 ± 3.90	33.6 ± 30.10	*Yr18*+*Yr29*
BW 9189	3+	3+	3+	3+	3+	66.7 ± 11.50	653.33 ± 106.90	*Yr29*
BW 16556	0;	0;	3+	0;	0;	0.7 ± 1.20	3.73 ± 6.50	*Yr18*
BW 16609	3	3	3+	0;	3+	66.7 ± 23.10	525 ± 181.90	0
BW 15845	3+	3+	3+	3+	3+	80 ± 0.00	770 ± 121.20	0
BW 16611	3+	3+	3	3+	0;	44 ± 31.70	333.67 ± 256.80	*Yr29*
BW 16612	3+	3+	3−	0;	3+	5.3 ± 9.20	42 ± 72.70	*Yr18*+*Yr29*
BW 16614	3+	3+	3+	3+	3+	38.7 ± 22.00	331.33 ± 262.00	*Yr18*
BW 16635	0;	0;	3+	0;	0;	25.3 ± 12.90	198.33 ± 102.00	*Yr29*
BW 15782	3+	3+	3+	3+	3+	53.3 ± 11.50	497 ± 163.10	*Yr29*
BW 15907	0;	0;	3+	3+	3+	58.7 ± 37.00	515.67 ± 384.40	*Yr29*
BW 15908	3	3	0;	3+	3+	64 ± 27.70	644 ± 339.50	*Yr29*
BW 16096	;3	;3	3	3+	3+	80 ± 0.00	770 ± 242.50	*Yr29*
BW 16098	3+	3+	3+	3+	3+	73.3 ± 11.50	688.33 ± 336.30	*Yr29*
BW 16099	3+	3+	3+	3	3+	45.3 ± 25.40	403.67 ± 270.80	*Yr29*
BW 16101	3+	3+	3+	3+	3+	46.7 ± 11.50	455 ± 273.40	*Yr29*
BW 16102	;3	;3	3−	3+	3+	60 ± 20.00	567 ± 263.10	0
BW 16103	3+	3+	3+	3+	3+	80 ± 0.00	683.67 ± 135.80	*Yr29*
BW 16104	3+	3+	3+	3+	3+	38.7 ± 22.00	269.5 ± 154.80	*Yr29*
BW 16106	3+	3+	3+	3+	3+	21.3 ± 16.70	155.63 ± 129.60	*Yr29*
BW 16107	3+	3+	3+	3+	3+	46.7 ± 11.50	415.33 ± 125.50	*Yr18*
BW 16108	3	3	3	0;	0;	18.7 ± 18.50	205.8 ± 129.90	*Yr18*
BW 16109	3+	3+	3+	3+	3+	73.3 ± 11.50	630 ± 0.00	*Yr29*
CWI 10963	3+	3+	3+	0;	3	60 ± 20.00	513.33 ± 145.70	0
BW 16112	3+	3+	3+	0;	3+	36 ± 38.60	281.63 ± 305.80	*Yr18*
BW 1896	3+	3+	3+	3+	3+	66.7 ± 11.50	597.33 ± 153.60	*Yr29*
BW2325	0;	0;	3	3+	0;	56 ± 41.60	525 ± 408.80	*Yr29*
BW 2327	3+	3+	3	3+	2−	53.3 ± 23.10	550.67 ± 260.20	*Yr29*
BW 2663	3+	3+	3+	3+	3+	60 ± 20.00	595 ± 264.20	*Yr29*
BW 6251	3	3	3−	3+	3+	46.7 ± 11.50	415.33 ± 125.50	*Yr18*
BW 8319	0;	0;	3+	3+	0;	22.7 ± 8.30	219.33 ± 155.30	*Yr18*+*Yr46*
BW 8320	3+	3+	3+	3+	0;	46.7 ± 23.10	385 ± 245.00	*Yr46*
BW 8321	3+	3+	3+	3+	3+	33.3 ± 11.50	298.67 ± 47.60	*Yr29*+*Yr46*
BW 8322	3+	3+	3+	3+	3+	16 ± 13.90	168.93 ± 193.20	*Yr18*+*Yr29*+*Yr46*
BW 8325	3+	3+	3+	3+	3−	16 ± 0.00	150.5 ± 41.30	*Yr18*+*Yr29*
BW 8334	3+	3+	3+	3+	0;	53.3 ± 11.50	555.33 ± 217.00	*Yr18*
BW 8335	0;	0;	3+	3+	0;	66.7 ± 11.50	571.67 ± 248.30	*Yr29*
BW 8336	0;	0;	3+	3+	0,	36 ± 26.20	312.67 ± 248.50	*Yr29*
BW 8337	3−	3−	3N	3+	2−	53.3 ± 30.60	452.67 ± 249.00	*Yr18*+*Yr29*
BW 8341	3	3	3+	3+	3+	60 ± 0.00	597.33 ± 157.40	*Yr29*
BW 8950	3−	3−	3	3+	0;	46.7 ± 11.50	485.33 ± 251.40	*Yr29*
BW 9242	3+	3+	3+	3+	0;	60 ± 0.00	487.67 ± 91.40	*Yr29*+*Yr46*
BW 12202	3+	3+	3+	3+	3+	49.3 ± 37.20	476 ± 371.20	*Yr29*+*Yr46*
BW 29910	0;	0;	3+	0;	;	49.3 ± 37.20	557.67 ± 493.50	*Yr29*+*Yr46*
T 3862	3+	3+	3+	3+	0;	49.3 ± 37.20	541.33 ± 441.00	*Yr29*+*Yr46*
CWI 36473	3	3	3+	3+	;−	13.3 ± 4.60	109.9 ± 78.40	*Yr46*
CWI 41172	3	3	3−	3−	3−	32 ± 41.60	322.7 ± 448.50	*Yr46*
CWI 32610	3+	3+	3+	3+	0;	66.7 ± 11.50	665 ± 344.70	*Yr29*
CWI 85928	0;	0;	3+	3+	;	40 ± 0.00	420 ± 121.20	*Yr29*+*Yr46*
BW 35109	3+	3+	3+	3	3+	0 ± 0.00	0 ± 0.00	*Yr18*+*Yr29*+*Yr46*
BW 35110	3+	3+	3+	3+	0;	0 ± 0.00	0 ± 0.00	*Yr18*+*Yr29*+*Yr46*
BW 35112	3+	3+	3+	3	3+	0 ± 0.00	0 ± 0.00	*Yr18*+*Yr29*+*Yr46*
BW 35114	3+	3+	3+	3+	3+	0 ± 0.00	0 ± 0.00	*Yr29*+*Yr46*
BW 35116	3+	3+	3+	3+	3+	0 ± 0.00	0 ± 0.00	*Yr29*+*Yr46*
DW 7958	3+	3+	3+	3+	3+	46.7 ± 11.50	443.33 ± 145.70	*Yr46*
DW 7963	0;	0;	3+	3+	0;	37.3 ± 4.60	317.33 ± 168.20	*Yr46*
BW 27274	3+	3+	3+	3+	0;	50.7 ± 25.70	445.67 ± 282.60	*Yr29*+*Yr46*
BW 33914	0;	0;	3+	3+	3+	46.7 ± 30.60	609 ± 388.90	*Yr46*
BW 32061	0;	0;	3+	3+	0;	40 ± 20.00	350 ± 124.40	*Yr18*+*Yr29*+*Yr46*
BW 37109	0;	0;	3+	3+	0;	60 ± 20.00	616 ± 379.00	*Yr29*+*Yr46*
CWI 51010	3+	3+	3+	3+	0;	44 ± 31.70	389.67 ± 208.90	*Yr46*
CWI 13085	3+	3+	3+	3+	0;	40 ± 34.60	415.33 ± 367.80	*Yr29*+*Yr46*
BW 44529	3+	3+	3+	3+	3+	53.3 ± 23.10	555.33 ± 428.40	*Yr29*+*Yr46*
BW 44334	3+	3+	3+	3+	3+	44 ± 14.40	462 ± 268.20	*Yr46*
BW 44335	3+	3+	3+	3+	3+	45.3 ± 32.30	429.33 ± 372.90	0
BW 44336	3+	3+	3+	3+	3+	60 ± 20.00	555.33 ± 280.10	*Yr46*
BW 44337	3+	3+	3+	3+	3+	53.3 ± 23.10	513.33 ± 291.40	*Yr46*
BW 44338	3+	3+	3+	3+	3+	40 ± 34.60	396.67 ± 385.50	*Yr46*
CWI 29059	0;	0;	3+	3+	3+	28 ± 6.90	219.33 ± 71.80	*Yr46*
CWI 7130	3+	3+	3+	3+	3+	46.7 ± 11.50	401.33 ± 138.10	*Yr29*+*Yr46*
CWI 8045	3	3	3	3+	3+	29.3 ± 18.50	242.67 ± 127.00	*Yr46*
CWI 9737	3+	3+	3	3+	3+	28 ± 28.00	264.13 ± 268.40	*Yr29*+*Yr46*
CWI 91687	3+	3+	3+	3+	3+	66.7 ± 23.10	625.33 ± 287.00	*Yr29*+*Yr46*
CWI 98580	3+	3+	3+	3+	3+	53.3 ± 11.50	529.67 ± 228.60	*Yr18*+*Yr46*
CWI 98601	3+	3+	3+	3+	3+	25.3 ± 12.90	179.67 ± 87.50	*Yr18*+*Yr29*+*Yr46*
CWI 98609	3+	3+	3+	3+	3+	44 ± 14.40	392 ± 328.00	*Yr18*+*Yr29*+*Yr46*
CWI 98629	3+	3+	3	3+	3+	50.7 ± 25.70	443.33 ± 343.60	*Yr29*+*Yr46*
CWI 98662	3+	3+	3+	3+	3+	22.7 ± 16.20	240.33 ± 222.50	YR29+YR46
CWI 75596	3+	3+	3+	3+	3+	49.3 ± 37.20	536.67 ± 409.90	*Yr18*+*Yr46*
CWI 98753	3+	3+	3+	3+	3+	53.3 ± 30.60	449.17 ± 351.90	*Yr29*+*Yr46*
CWI 98815	3+	3+	3+	3+	3+	50.7 ± 25.70	464.33 ± 392.90	*Yr18*+*Yr46*
CWI 98817	2−	2−	3−	;N	3	36 ± 26.20	345.8 ± 331.40	*Yr29*+*Yr46*
CWI 98886	0;	0;	2−	2N	;−	40 ± 20.00	378 ± 285.90	*Yr18*+*Yr29*+*Yr46*
CWI 98913	3+	3+	3+	3+	3+	21.3 ± 18.00	242.67 ± 103.20	*Yr18*+*Yr29*+*Yr46*
CWI 98928	3+	3+	3+	3+	3+	46.7 ± 23.10	494.67 ± 313.20	*Yr46*
BW 8947	3+	3+	3+	3+	3+	53.3 ± 11.50	476 ± 91.80	*Yr18*+*Yr29*+*Yr46*
CWI 99449	0;	0;	0;	0;	0;	26.7 ± 30.50	276.27 ± 321.20	*Yr29*+*Yr46*
CWI 99459	3+	3+	3+	3+	3+	66.7 ± 23.10	616 ± 301.20	*Yr46*
BW 15906	3+	3+	3+	3+	3+	36 ± 26.20	326.67 ± 204.00	*Yr46*
CWI 99812	3+	3+	3+	;N	3+	46.7 ± 11.50	406 ± 77.90	*Yr18*+*Yr46*
CWI 41166	2−	2−	0;	0;	0;	12 ± 6.90	107.33 ± 76.80	*Yr18*+*Yr46*
T 1074	;N	;N	;N	3+	3+	18.7 ± 18.50	130.67 ± 117.20	*Yr46*
CWI 41396	3+	3+	3+	3+	3−	34.7 ± 39.50	284.67 ± 299.40	*Yr46*
T 1337	3+	3+	3+	3+	3−	32 ± 41.60	250.13 ± 329.00	*Yr46*
CWI 41399	3−	3−	3+	3+	3−	14.7 ± 6.10	179.67 ± 150.80	*Yr29*+*Yr46*
T 1659	3+	3+	3+	3+	3−	36 ± 26.20	280 ± 188.00	*Yr29*+*Yr46*
DW 895	3+	3+	3+	3+	3+	42.7 ± 36.10	387.8 ± 409.10	*Yr46*
19th HTWYT2	;	;	2−	3+	3+	6.7 ± 2.30	46.2 ± 24.40	*Yr29*+*Yr46*
19th HTWYT3	0;	0;	3+	3+	2−	9.3 ± 6.10	66.27 ± 52.20	*Yr29*+*Yr46*
19th HTWYT4	3−	3−	3+	3+	3−	21.3 ± 9.20	163.33 ± 102.00	*Yr46*
19th HTWYT5	0;	0;	3+	3+	0;	42.7 ± 4.60	345.33 ± 130.10	0
19th HTWYT6	0;	0;	3+	3+	3+	18.7 ± 12.20	144.67 ± 118.00	*Yr46*
19th HTWYT7	0;	0;	3+	3+	2−	16 ± 13.90	135.33 ± 125.30	*Yr29*+*Yr46*
19th HTWYT8	0;	0;	3+	3+	3+	4.7 ± 3.10	46.2 ± 45.80	*Yr18*+*Yr29*+*Yr46*
19th HTWYT9	3+	3+	3+	3+	3+	12 ± 6.90	121.8 ± 106.70	*Yr18*+*Yr29*+*Yr46*
19th HTWYT10	2−	2−	3+	3+	0;	32 ± 24.30	263.67 ± 78.70	*Yr29*+*Yr46*
41st ESWYT 111	3−	3−	3+	3+	3+	34.7 ± 39.50	343 ± 431.60	*Yr46*
41st ESWYT 112	0;	0;	0;	3+	0;	2.9 ± 4.40	23.57 ± 34.20	*Yr18*+*Yr46*
41st ESWYT 113	3	3	3+	3	3+	4.1 ± 3.90	33.6 ± 27.60	*Yr46*
41st ESWYT 114	0;	0;	0;	0;	2−	9.3 ± 6.10	70.7 ± 60.40	*Yr29*+*Yr46*
41st ESWYT 115	3+	3+	3+	3+	3+	10.7 ± 4.60	82.13 ± 38.10	*Yr29*+*Yr46*
41st ESWYT 116	0;	0;	3+	0;	0;	25.3 ± 30.00	216.3 ± 297.90	*Yr46*
41st ESWYT 117	0;	0;	0;	3+	0;	32 ± 41.60	331.8 ± 440.60	*Yr18*+*Yr29*+*Yr46*
41st ESWYT 118	0;	0;	3+	3+	3+	22.7 ± 16.20	178.5 ± 127.30	*Yr18*+*Yr29*+*Yr46*
41st ESWYT 119	0;	0;	0;	0;	0;	28.1 ± 45.00	286.77 ± 479.20	*Yr29*+*Yr46*
41st ESWYT 120	;	;	3+	3+	3+	34.7 ± 39.50	413 ± 552.60	*Yr18*+*Yr29*+*Yr46*
51st ESWYT 121	0;	0;	0;	3+	0;	34.7 ± 39.50	399.47 ± 563.60	*Yr18*+*Yr29*+*Yr46*
51st ESWYT 122	0;	0;	3+	3+	0;	21.3 ± 16.70	170.33 ± 93.00	*Yr29*+*Yr46*
51st ESWYT 123	0;	0;	3+	3+	0;	42.7 ± 36.10	476 ± 512.80	*Yr29*+*Yr46*
51st ESWYT 124	0;	0;	3+	3+	0;	42.7 ± 36.10	469.47 ± 520.80	*Yr18*+*Yr29*
51st ESWYT 125	3+	3+	3+	3+	3+	30 ± 24.60	264.83 ± 247.10	*Yr29*+*Yr46*
51st ESWYT 126	3+	3+	3+	3+	0;	28 ± 20.80	219.33 ± 154.40	*Yr29*+*Yr46*
51st ESWYT 127	0;	0;	3	3+	3−	18.7 ± 18.50	170.33 ± 156.60	*Yr29*+*Yr46*
51st ESWYT 128	;−	;−	0;	0;	0;	66.7 ± 23.10	625.33 ± 287.00	*Yr18*+*Yr29*+*Yr46*
51st ESWYT 129	;−	;−	0;	0;	0;	53.3 ± 11.50	529.67 ± 228.60	*Yr18*+*Yr46*
51st ESWYT 130	3+	3+	3+	3+	3+	22.7 ± 16.20	184.33 ± 139.00	ND
51st ESWYT 131	0;	0;	2	3+	0;	22.7 ± 16.20	177.8 ± 143.10	*Yr29*
51st ESWYT 132	0;	0;	3+	3+	0;	40 ± 20.00	340.67 ± 205.00	*Yr29*
51st ESWYT 133	0;	0;	3+	3+	0;	33.3 ± 11.50	301 ± 178.60	*Yr46*
51st ESWYT 134	;N	;N	3−	3+	0;	6 ± 3.5.00	50.87 ± 43.90	*Yr29*+*Yr46*
51st ESWYT 135	;−	;−	;	0;	0;	0.2 ± 0.00	2.8 ± 0.00	*Yr29*+*Yr46*
51st ESWYT 136	0;	0;	0;	3−	0;	8 ± 0.00	72.57 ± 22.30	*Yr18*+*Yr29*+*Yr46*
51st ESWYT 137	0;	0;	3+	3+	0;	26.7 ± 11.50	280 ± 183.10	*Yr29*
51st ESWYT 138	0;	0;	3+	3	0;	23.3 ± 15.30	261.33 ± 124.50	*Yr46*
51st ESWYT 139	0;	0;	3+	3+	3+	9.3 ± 9.20	91 ± 91.50	*Yr46*
51st ESWYT 140	3−	3−	3+	0;	3−	0.1 ± 0.10	494.67 ± 313.20	ND
51st ESWYT 141	3+	3+	3+	3+	0;	4.1 ± 3.90	43.4 ± 49.10	*Yr18*+*Yr29*+*Yr46*
51st ESWYT 142	3+	3+	3+	1N	3	6.7 ± 2.30	45.03 ± 17.20	*Yr29*+*Yr46*
51st ESWYT 143	3	3	3+	3+	3+	10.7 ± 8.30	102.67 ± 81.10	*Yr29*+*Yr46*
51st ESWYT 144	0;	0;	3+	3+	3+	17.3 ± 19.70	196 ± 255.50	*Yr18*+*Yr29*+*Yr46*
51st ESWYT 145	;	;	0;	0;	;	0 ± 0.00	0 ± 0.00	*Yr29*+*Yr46*
51st ESWYT 146	0;	0;	0;	0;	0;	0 ± 0.00	0 ± 0.00	*Yr29*+*Yr46*
51st ESWYT 147	0;	0;	;−	0;	0;	0 ± 0.00	0 ± 0.00	*Yr29*+*Yr46*
51st ESWYT 148	3	3	3−	3+	3+	29.3 ± 20.10	266 ± 175.40	*Yr29*+*Yr46*
51st ESWYT 149	;	;	0;	3+	2−	28 ± 17.40	261.33 ± 167.00	*Yr29*+*Yr46*

Infection types follow the 0–4 scale of Stakman et al. [18] as modified by Nayar et al. [19] and Bhardwaj et al. [20] and are shown in Table S2. Infection types were classified as follows: 0 = no visible uredinia; − (fleck minus) = slight necrosis/micro-flecking visible; 0; and ; (naught fleck and fleck) = no uredinia but hypersensitive flecks present; N = necrotic area without sporulation; 1 = necrotic and chlorotic areas with restricted sporulation; 2 = moderate sporulation with necrosis and chlorosis; 3 = sporulation with chlorosis; 3+ (three plus) = abundant sporulation without chlorosis; X = variable types of uredinia; Y = susceptible types of uredinia at the leaf tip and resistant types toward the leaf base; and Z = resistant types of uredinia at the leaf tip and susceptible types toward the leaf base. MAS, marker-assisted selection using SSR markers *csLV34* (*Yr18*), *wmc44* (*Yr29*) and *gwm192* (*Yr46*).

**Table 3:** Summary of molecular markers used for molecular postulation of the APR genes *Yr18,Yr29* and *Yr46* in the present study.

Marker	Target Gene	Marker Type	Chromosome Location	Distance to Gene (cM)	Amplicon Size (bp)	Reference
*csLV34*	*Yr18* (*Lr34*)	Gene-derived, co-dominant STS marker	7DS	0.4 (within the gene haplotype)	150 (resistant allele)	[51]
*wmc44*	*Yr29* (*Lr46*)	Genomic SSR	1BL	~3.6 proximal	260	[52]
*gwm192*	*Yr46* (*Lr67*)	Genomic SSR	4DL	0.4 proximal	130	[53]

## Data Availability

The datasets generated and analysed for this study are included in the article and its Supplementary Materials. Further inquiries can be directed to the corresponding author.

## Supplementary Materials

The following supporting information can be downloaded at: https://www.mdpi.com/article/10.3390/plants15213315/s1.
