# Supplementary material for: Phenotypic and Molecular Characterization of Adult Plant Resistance to Stripe Rust (Puccinia striiformis f. sp. tritici) in Exotic and Indigenous Wheat Germplasm
Source: Plants (Basel). 2026 Oct 26;15(21):3315. doi: 10.3390/plants15213315 (PMC13765799; doi:10.3390/plants15213315)
Supplement: Supplementary file 1 [file plants-15-03315-s001.zip › Supplementary Figure.pdf]

Figure S1. Molecular identification of *Yr18* gene among the wheat genotypes

The presence of the yellow rust resistance gene *Yr*18 was determined using the STS marker csLV34. This marker located 0.4 cM distal to *Yr*18 and an amplicon of 150 bp indicated the presence of the *Yr18* gene In this study, PCR analysis confirmed the association of 66 wheat genotypes (33.5%) with the *Yr18* gene.

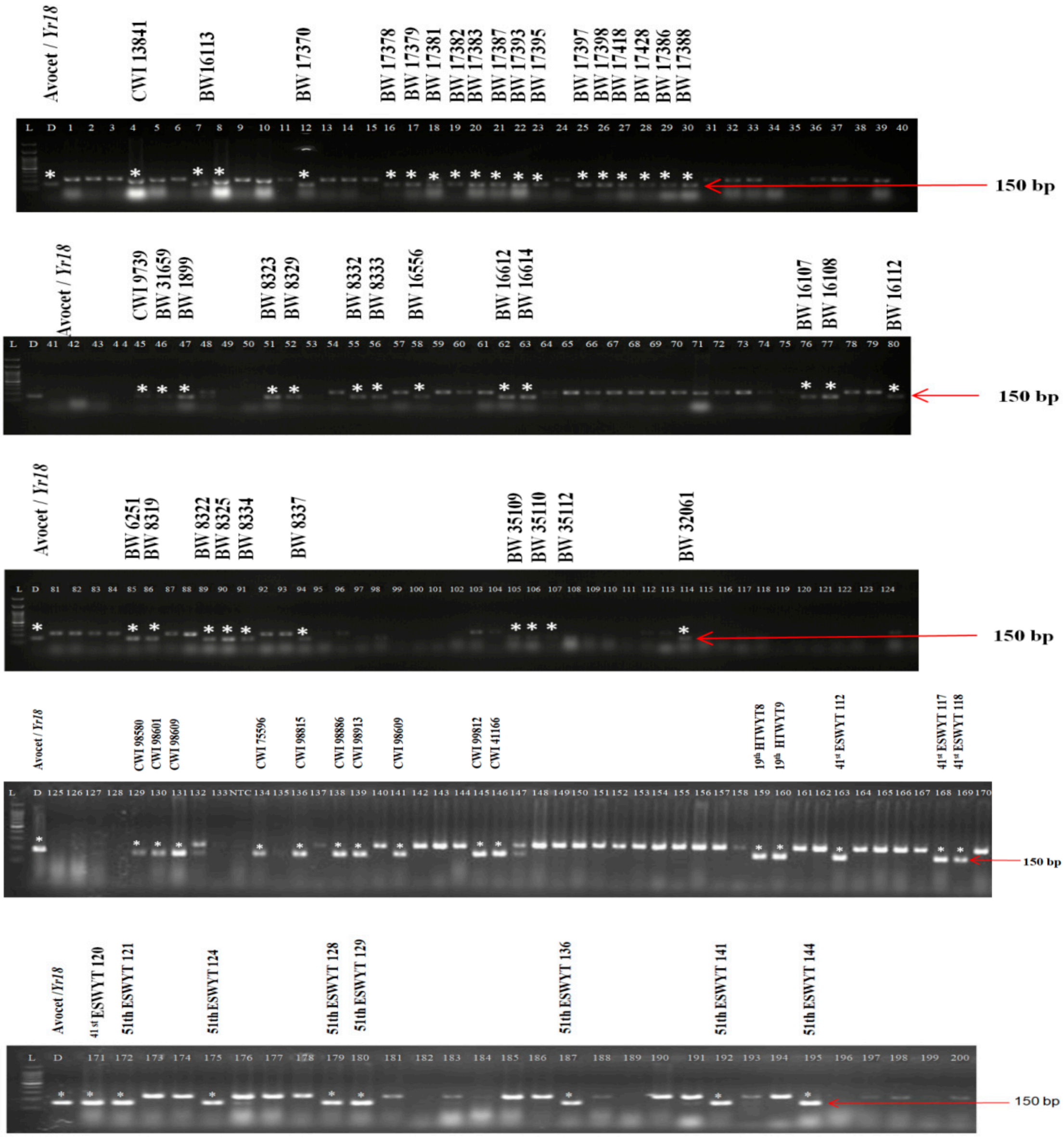

Figure S1. Amplification of *Yr*18 genes among the wheat germplasm lines

Figure S2. Molecular identification of *Yr29* gene among the wheat genotypes

Yr29, initially identified in the cultivar Pavon 76, is located on chromosome 1BL. This gene provides a moderate level of slow-rusting adult plant resistance (APR) to yellow rust and is closely associated with *Lr46*, which imparts resistance to leaf rust. The molecular marker Wmc44, situated 3.6 cM proximal to *Yr29,* amplified a 260 bp allele. This allele is present in 68% of genotypes, totaling 136 entries**.**

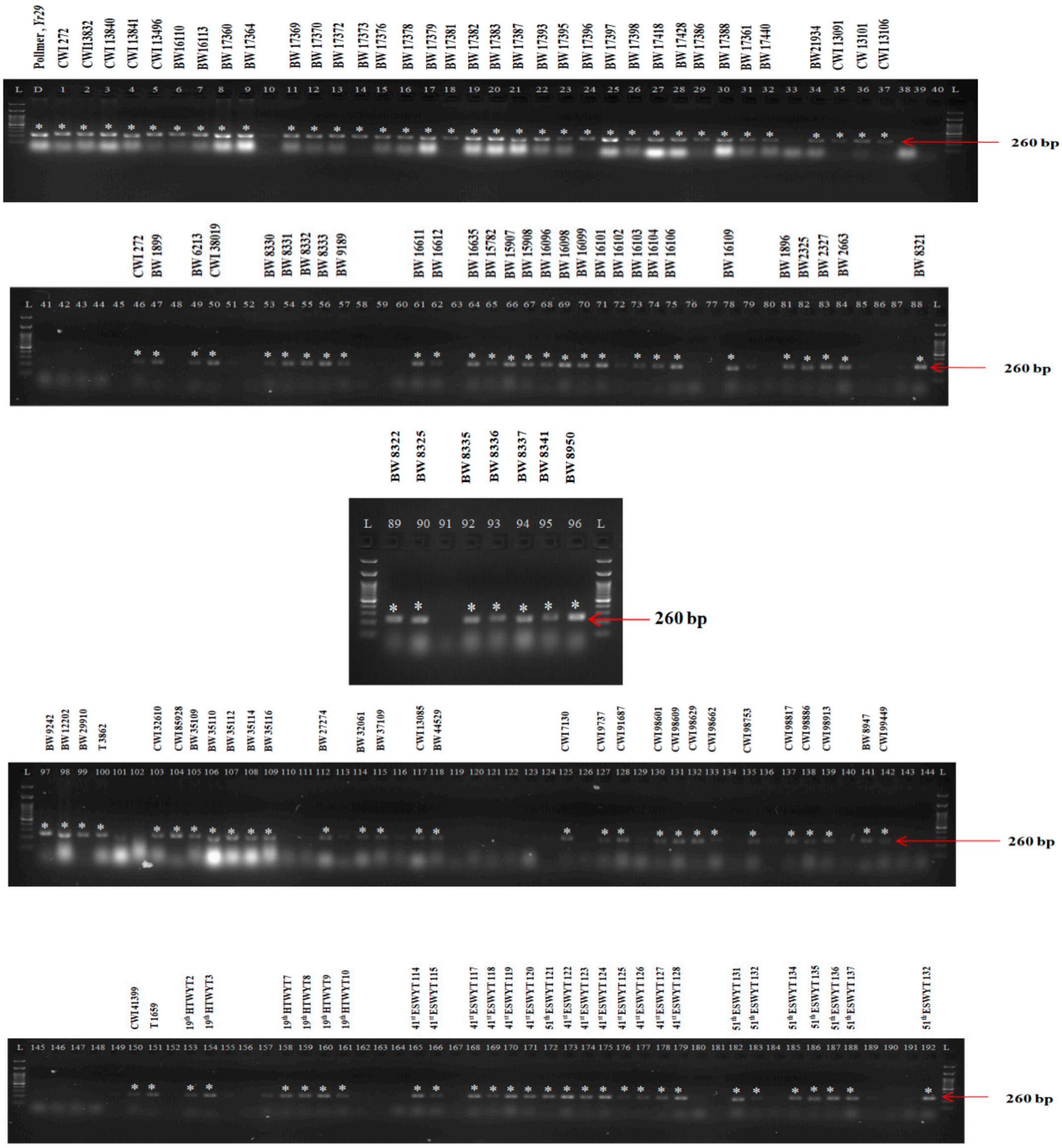

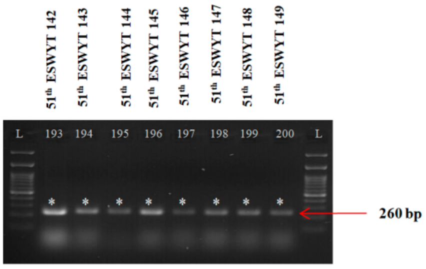

Figure S2. PCR amplification of *Yr*29 genes among the tested germplasm lines

Figure S3. Molecular identification of *Yr*46 genes among the tested germplasm lines

*Yr*46, located in the middle of chromosome 4DL, provides adult plant resistance (APR) to yellow rust. The marker *gwm*192, positioned 0.4 cM proximally to the *Yr*46 gene, amplifies a single 130 bp allele (+) in 124 genotypes. Among the genotypes, 62% genotypes specifically amplified the 130 bp allele associated with *Yr46*.

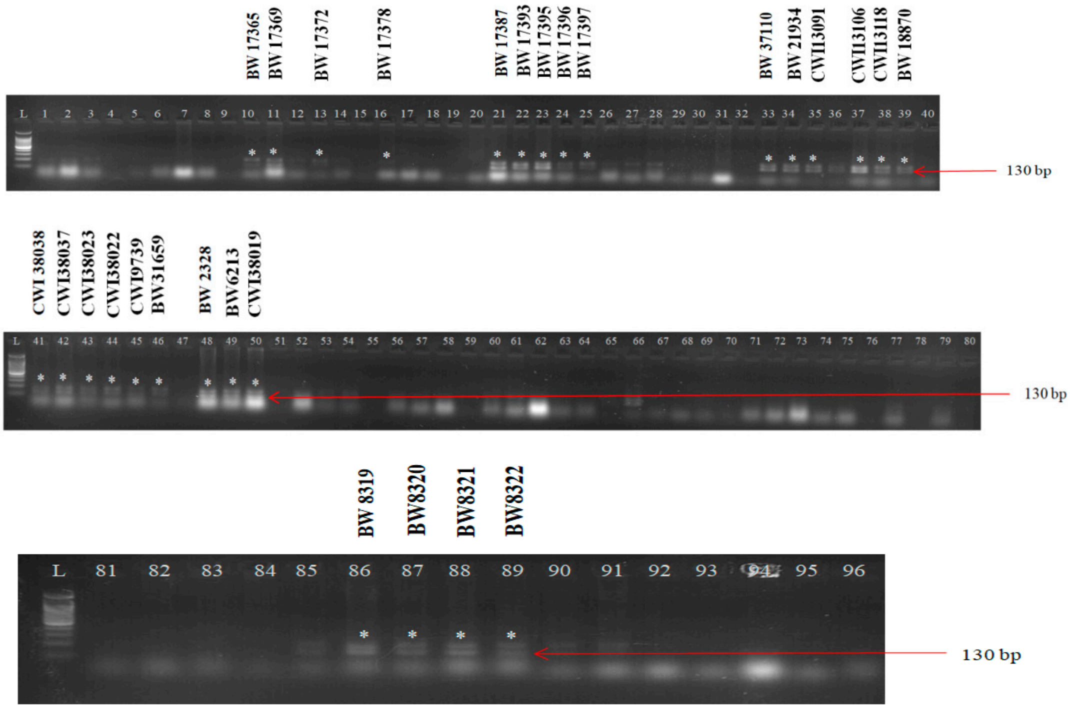

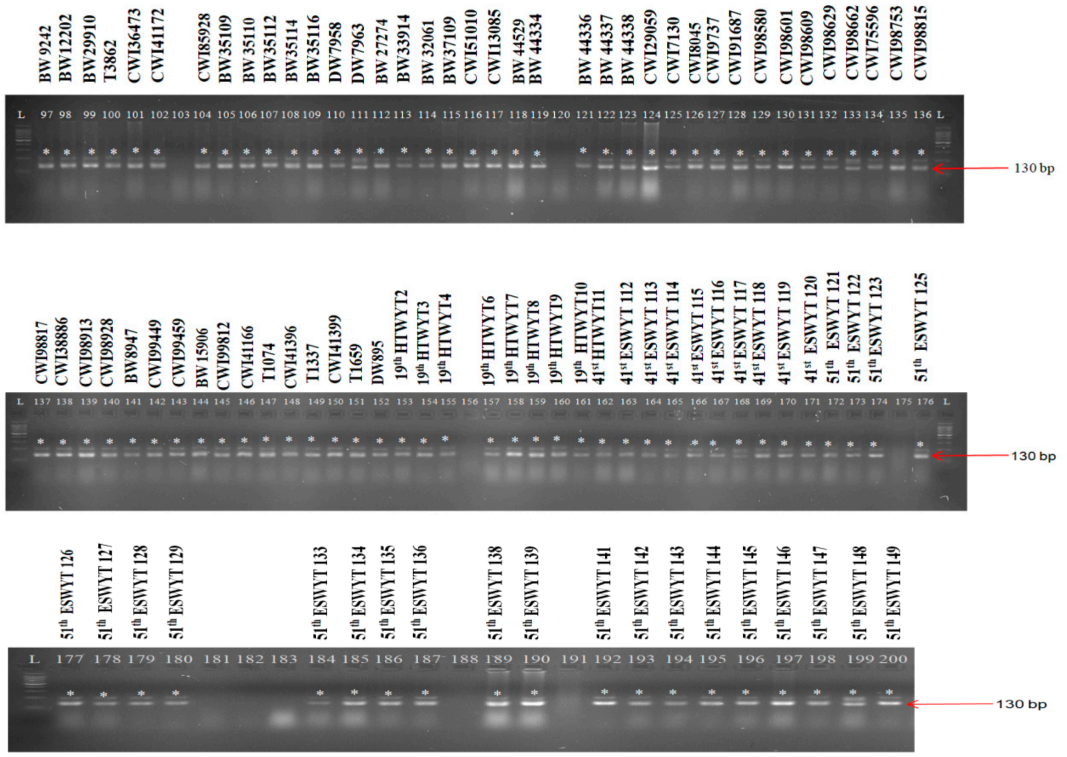

Figure S3. PCR based Amplification of *Yr 46* genes with tagged markers (*gwm192*) among the tested wheat germplasm lines and their allele sizes (bp)
