# Supplementary material for: Phenotypic and Molecular Characterization of Adult Plant Resistance to Stripe Rust (Puccinia striiformis f. sp. tritici) in Exotic and Indigenous Wheat Germplasm
Source: Plants (Basel). 2026 Oct 26;15(21):3315. doi: 10.3390/plants15213315 (PMC13765799; doi:10.3390/plants15213315)
Supplement: Supplementary file 1 [file plants-15-03315-s001.zip › Supplementary table.pdf]

Table S1. List of the 200 wheat genotypes evaluated in this study, with their pedigree.

Genotype	Pedigree
CWI 272	JAL UP
CWI13832	CItr 7278
CWI 13840	LYALLPUR-CRONOCO-III
CWI 13841	LYALLPUR-CRONOCO-II
CWI 13496	CItr 4907
BW16110	GID 28557
BW16113	GID 284661
BW 17360	CPAN2002
BW 17364	HD2405
BW 17365	PAU.ACC.3157-?
BW 17369	WG3455
BW 17370	WL3399
BW 17372	WL4547
BW 17373	WL6572
BW 17376	WL6737
BW 17378	WL7060
BW 17379	WL7104
BW 17381	WL7290
BW 17382	WL7349
BW 17383	W30
BW 17387	W341
BW 17393	W531
BW 17395	W573
BW 17396	W1106
BW 17397	W1320-F4
BW 17398	W2267
BW 17418	W1095
BW 17428	WL7273
BW 17386	W231
BW 17388	W381
BW 17361	EC165634
BW 17440	W499
BW37110	-0IND
BW21934	-0M
CWI 13091	W-63
CW 13101	W-73
CWI 13106	W-78-A
CWI 13118	W-91
BW 18870	CPAN2016
CWI 38039	CWI16912-3Y
CWI 38038	CWI16912-2Y
CWI 38037	CWI16912-1Y
CWI 38023	CWI17071-4Y

Genotype	Pedigree
CWI 38022	CWI17071-3Y
CWI 9739	HARLAN J.R 989
BW 31659	-0IND
BW 1899	J43-2L
BW 2328	IWP85
BW 6213	JT35
CWI 38019	CWI17048-5Y
BW 8323	BW-14
BW 8329	BW-20
BW 8330	BW-21
BW 8331	BW-22
BW 8332	BW-24
BW 8333	BW-25
BW 9189	HOPPS
BW 16556	WL 6736
BW 16609	LOK BHARATI 2
BW 15845	LOK BHARATI 3
BW 16611	MP-844
BW 16612	GID 60322
BW 16614	GID 45055
BW 16635	HYBRID DELHI 29
BW 15782	W203
BW 15907	W 1301
BW 15908	WG 3070
BW 16096	GID 28504
BW 16098	GID 28514
BW 16099	GID 28517
BW 16101	GID 28521
BW 16102	GID 28525
BW 16103	GID 28532
BW 16104	GID 28538
BW 16106	GID 28544
BW 16107	GID 31505
BW 16108	GID 31511
BW 16109	GID 28549
CWI 10963	NP-789
BW 16112	GID 284660
BW 1896	JT35-2L
BW2325	HD-2167
BW 2327	DEBEIRA
BW 2663	HD 2162
BW 6251	JT43
BW 8319	BW-7
BW 8320	BW-8
BW 8321	BW-9
BW 8322	BW-10

Genotype	Pedigree
BW 8325	BW-16
BW 8334	BW-26
BW 8335	BW-28
BW 8336	BW-31
BW 8337	BW-32
BW 8341	BW-44
BW 8950	HYBRID DELHI 832
BW 9242	GID 272325
BW 12202	HYBRID DELHI 2327
BW 29910	-0IND
T 3862	TL1632
CWI 36473	UPT79340
CWI 41172	JNK6T0150
CWI 32610	AP425
CWI 85928	AJEBA 210
BW 35109	W18-3-2
BW 35110	W18-7-4
BW 35112	W18-10-2
BW 35114	W18-15-3
BW 35116	W18-20-1
DW 7958	BYC.651
DW 7963	YARASU.1.8
BW 27274	-0IND
BW 33914	-(JAGUISH)-0IND
BW 32061	-(RAJLAXMIN)-0IND
BW 37109	-0IND
CWI 51010	CB (BW) 362
CWI 13085	W-40
BW 44529	-0IND-01Y
BW 44334	IG 130968
BW 44335	IG 130973
BW 44336	IG 130974
BW 44337	IG 130976
BW 44338	IG 131004
CWI 29059	RL5
CWI 7130	KOELZ W 11193
CWI 8045	INDISKT
CWI 9737	DIRK
CWI 91687	HARD RED CALCUTTA
CWI 98580	RED TIKORIA
CWI 98601	WAT-1190124
CWI 98609	FEDERATION 2
CWI 98629	THORI
CWI 98662	SHAN-WHEAT
CWI 75596	T.DICOCCON DWARF
CWI 98753	GAJAR

Genotype	Pedigree
CWI 98815	SPIN
CWI 98817	WAT-1190520
CWI 98886	KAGHZI DESI
CWI 98913	WALAITI
CWI 98928	PUSA-62
BW 8947	HYBRID DELHI 1944
CWI 99449	IG 43518
CWI 99459	IG 43545
BW 15906	W 237
CWI 99812	IG 40605
CWI 41166	UC8825
T 1074	JNK6T090
CWI 41396	TL17
T 1337	TL22
CWI 41399	JNK6T059
T 1659	TL15
DW 895	RABI
19th HTWYT2	NADI #1
19th HTWYT3	WBLL1*2/BRAMBLING/4/BABAX/LR42//BABAX*2/3/SHAMA*2/5/PBW343*2/KUKUNA*2//FRTL/PIFED
19th HTWYT4	QUAIU #1/SUP152
19th HTWYT5	PBW343*2/KUKUNA/3/PASTOR//CHIL/PRL/4/GRACK/5/MUU/FRNCLN//FRANCOLIN #1
19th HTWYT6	CHIBIA//PRLII/CM65531/3/FISCAL*2/4/TAM200/TURACO/5/SWSR22T.B./*2BLOUK #1//WBLL1*2/KURUKU
19th HTWYT7	WBLL1*2/BRAMBLING*2//BAVIS/3/CHYAK1/VILLA JUAREZ F2009//WBLL1*2/BRAMBLING
19th HTWYT8	KACHU//WBLL1*2/BRAMBLING*2/6/BECARD #1/5/KIRITATI/4/2*SERI.1B*2/3/KAUZ*2/BOW//KAUZ
19thHTWYT 9	SUP152/BAJ #1*2/3/KINGBIRD #1//INQALAB 91*2/TUKURU
19thHTWYT 10	SUP152/BAJ #1*2/3/KINGBIRD #1//INQALAB 91*2/TUKURU
41st ESWYT 111	WBLL1*2/BRAMBLING*2//BAVIS/3/BORL14
41 ESWYT 112	KENYA SUNBIRD/2*KACHU/3/SWSR22T.B./*2BLOUK #1//WBLL1*2/KURUKU
41 ESWYT 113	PFAU/MILAN/5/CHEN/AEGILOPS SQUARROSA (TAUS)//BCN/3/VEE#7/BOW/4/PASTOR/6/2*BAVIS #1/7/BORL14
41 ESWYT 114	MUCUY*2//SUP152/BAJ #1
41 ESWYT 115	ABLEU*2//KACHU/DANPHE
41 ESWYT 116	WBLL1*2/CHAPIO/6/CNDO/R143//ENTE/MEXI75/3/AE.SQ/4/2*OCI/5/2*CIRCUS/7/WBLL1*2/BRAMBLING*2/8/MUU/FRNCLN//FRANCOLIN #1
41 ESWYT 117	ND643/2*TRCH//BECARD/3/BECARD*2/4/MUU/FRNCLN
41 ESWYT 118	HEILO//MILAN/MUNIA/3/KIRITATI/2*TRCH/4/2*KACHU/KIRITATI

Genotype	Pedigree
41 ESWYT 119	MUTUS*2/HARIL #1/3/SWSR22T.B./2*BLOUK #1//WBLL1*2/KURUKU/4/MUTUS*2/HARIL #1
41 ESWYT 120	WHEAR/KUKUNA/3/C80.1/3*BATAVIA//2*WBLL1*2/4/KENYA SUNBIRD*2/5/ABLEU
51th ESWYT 121	MUTUS*2//ND643/2*WBLL1/3/2*SWSR22T.B./KACHU//2*KACHU
51th ESWYT 122	SUP152*2/TINKIO #1/4/FRET2*2/SHAMA//KIRITATI/2*TRCH/3/BAJ #1/5/SUP152*2/TINKIO #1
51th ESWYT 123	SWSR22T.B./FRANCOLIN #1//2*FRNCLN/5/WHEAR/KUKUNA/3/C80.1/3*BATAVIA//2*WBLL1*2/4/NIINI #1/6/BORL14
51th ESWYT 124	KACHU*2/3/ND643//2*PRL/2*PASTOR/4/2*KACHU/DANPHE
51th ESWYT 125	QUAIU/MUNAL//QUAIU #2/3/SUP152/BAJ #1
51th ESWYT 126	PAURAQ/NELOKI/3/WBLL1*2/BRAMBLING*2//BAVIS
51th ESWYT 127	KACHU/DANPHE*2//KENYA SUNBIRD/KACHU
51th ESWYT 128	BAVIS/3/ATTILA/BAV92//PASTOR/5/CROC_1/AE.SQUARROSA (205)//BORL95/3/PRL/SARA//TSI/VEE#5/4/FRET2*2/6/SWSR22T.B./2*BLOUK #1//WBLL1*2/KURUKU
51th ESWYT 129	MUTUS/AKURI #1//MUTUS/3/KACHU #1/KIRITATI//KACHU
51th ESWYT 130	MUNAL*2/WESTONIA/4/KACHU/3/WHEAR//2*PRL/2*PASTOR
51th ESWYT 131	SWSR22T.B.//TACUPETO F2001*2/BRAMBLING/3/2*TACUPETO F2001*2/BRAMBLING/4/BORL14
51th ESWYT 132	BAVIS #1//ND643/2*WBLL1/3/BOKOTA
51th ESWYT 133	BECARD//KIRITATI/2*TRCH/3/BECARD/4/DANPHE/BAJ #1
51th ESWYT 134	KENYA SUNBIRD/KACHU/4/KACHU/3/WHEAR//2*PRL/2*PASTOR
51th ESWYT 135	MUTUS*2/HARIL #1/3/MUTUS//ND643/2*WBLL1
51th ESWYT 136	WBLL1*2/SHAMA//KACHU/3/CIRO16/4/COPIO
51th ESWYT 137	KACHU/SAUAL/4/ATTILA*2/PBW65//PIHA/3/ATTILA/2*PASTOR/5/KINGBIRD #1//INQALAB 91*2/TUKURU
51th ESWYT 138	BORL14*2/3/BAVIS*2//ATTILA*2/PBW65
51th ESWYT 139	NADI*2/3/SWSR22T.B./2*BLOUK #1//WBLL1*2/KURUKU
51th ESWYT 140	SUP152/BAJ #1*2/4/WHEAR/VIVITSI//WHEAR/3/PANDORA
51th ESWYT 141	BECARD/FRNCLN//2*BORL14
51th ESWYT 142	BECARD/FRNCLN//2*BORL14
51th ESWYT 143	WBLL1*2/BRAMBLING*2//BAVIS/3/BORL14
51th ESWYT 144	BAJ #1/KISKADEE #1//KACHU/KIRITATI/3/BAJ #1/KISKADEE #1
51th ESWYT 145	MUTUS*2//TAM200/TURACO*2/3/KFA/2*KACHU

Genotype	Pedigree
51th ESWYT 146	MUTUS2//TAM200/TURACO2/3/ROLF07*2/SHORTENED SR26 TRANSLOCATION
51th ESWYT 147	MUTUS2//TAM200/TURACO2/3/WBLL12/BRAMBLING2//BAVIS
51th ESWYT 148	QUAIU #1/BECARD//2*BORL14
51th ESWYT 149	MUTUS2/HARIL #12/3/SWSR22T.B./2BLOUK #1//WBLL12/KURUKU

Table S2. Infection type (IT) response classes used to score seedling reaction to stripe rust pathotypes.

Reaction type / grade	Category	Visible symptoms
0	Immune	No visible uredinia
;- (fleck minus)	Nearly Immune	Slight necrosis/micro flecking visible
0; and ; (naught fleck and fleck)	Very resistant	No uredinia but hypersensitive flecks present
N	Resistant	Necrotic area without sporulation
1 (one)	Resistant	Necrotic and chlorotic areas with restricted sporulation
2 (two)	Moderately Resistant	Moderate sporulation with necrosis and chlorosis
3 (three)	Moderately Susceptible	Sporulation with chlorosis
3+/4 (three plus/four)	Susceptible	Abundant sporulation without chlorosis
33+ (three three plus)	Highly Susceptible	Uredinia profusely sporulating
X	Heterogeneous	Variable types of uredinia
Y	Heterogeneous	Susceptible types of uredinia at the tip and resistant towards the base of leaf
Z	Heterogeneous	Resistant types of uredinia at the tip and susceptible types towards the leaf base

Scale after Stakman et al. (1962), as modified by Nayar et al. (1997) and Bhardwaj et al. (2010).
